# Influence of socio-economic, demographic and climate factors on the regional distribution of dengue in the United States and Mexico

**DOI:** 10.1186/s12942-020-00241-1

**Published:** 2020-11-02

**Authors:** Matthew J. Watts, Panagiota Kotsila, P. Graham Mortyn, Victor Sarto i Monteys, Cesira Urzi Brancati

**Affiliations:** 1 Institute of Environmental Science and Technology (ICTA), Autonomous University of Barcelona (UAB), Bellaterra, Spain; 2 Joint Research Centre (JRC-Seville), European Commission, Seville, Spain; 3 Servei de Sanitat Vegetal, DARP, Generalitat de Catalunya, Av. Meridiana, 38, 08018 Barcelona, Spain; 4 Barcelona Laboratory for Urban Environmental Justice and Sustainability (BCNEJ), Institute of Environmental Science and Technology (ICTA), Autonomous University of Barcelona (UAB), Bellaterra, Spain; 5 Department of Geography, Autonomous University of Barcelona (UAB), Bellaterra, Spain

**Keywords:** Dengue, Climate-change, Global-warming, Socio-economic, Mosquito-borne, Vector-borne-diseases, GDP, GAM

## Abstract

**Background:**

This study examines the impact of climate, socio-economic and demographic factors on the incidence of dengue in regions of the United States and Mexico. We select factors shown to predict dengue at a local level and test whether the association can be generalized to the regional or state level. In addition, we assess how different indicators perform compared to per capita gross domestic product (GDP), an indicator that is commonly used to predict the future distribution of dengue.

**Methods:**

A unique spatial-temporal dataset was created by collating information from a variety of data sources to perform empirical analyses at the regional level. Relevant regions for the analysis were selected based on their receptivity and vulnerability to dengue. A conceptual framework was elaborated to guide variable selection. The relationship between the incidence of dengue and the climate, socio-economic and demographic factors was modelled via a Generalized Additive Model (GAM), which also accounted for the spatial and temporal auto-correlation.

**Results:**

The socio-economic indicator (representing household income, education of the labour force, life expectancy at birth, and housing overcrowding), as well as more extensive access to broadband are associated with a drop in the incidence of dengue; by contrast, population growth and inter-regional migration are associated with higher incidence, after taking climate into account. An ageing population is also a predictor of higher incidence, but the relationship is concave and flattens at high rates. The rate of active physicians is associated with higher incidence, most likely because of more accurate reporting. If focusing on Mexico only, results remain broadly similar, however, workforce education was a better predictor of a drop in the incidence of dengue than household income.

**Conclusions:**

Two lessons can be drawn from this study: first, while higher GDP is generally associated with a drop in the incidence of dengue, a more granular analysis reveals that the crucial factors are a rise in education (with fewer jobs in the primary sector) and better access to information or technological infrastructure. Secondly, factors that were shown to have an impact of dengue at the local level are also good predictors at the regional level. These indices may help us better understand factors responsible for the global distribution of dengue and also, given a warming climate, may help us to better predict vulnerable populations on a larger scale.

## Introduction

The dengue virus (DENV) is one of the most important mosquito-borne viral diseases in the world today. Two main arthropod vectors are responsible for transmission of dengue viruses: *Aedes aegypti* (commonly known as yellow fever mosquito) and *Aedes albopictus* (commonly known as tiger mosquito). *A. aegypti* mainly feeds on humans and is highly adapted to human habitations and urban areas; *A. albopictus* feeds on animals and humans and is more prevalent in rural and peri-urban environments. While *A. albopictus* is also responsible for dengue transmission among humans, it is a less likely vector than *A. aegypti* since it is adapted to a wider range of environments and has less restrictive feeding habits [1]. Both *Aedes* mosquitoes are highly adapted to breeding in aquatic habitats like ponds and lakes, but also micro habitats, such as tree-holes, rock crevices and even leaf axils [2]. The latter behaviour in recent times has benefited both species by allowing them to exploit a range of man-made aquatic breeding habitats, where water can accumulate, like urban gardens, vases in cemeteries, discarded bottles and plant pots; therefore, both species can survive in drier climates than expected, by exploiting artificial water sources.

Dengue is a disease caused by any one of four closely related viruses: DENV 1, DENV 2, DENV 3, or DENV 4. Currently, all four dengue serotypes are in circulation in the Americas and can co-circulate within a region; the actual distribution of each serotype is difficult to establish for a number of reasons, such as inadequate surveillance, under reporting, high numbers of asymptomatic carriers, and so on, as laid out by [3]. DENV causes an acute flu-like illness that affects people of all age groups. Those who recover from a dengue infection can expect lifelong immunity against that serotype and some partial, but temporary, cross-immunity to the other serotypes, although secondary infections by other serotypes increase the risk of developing severe dengue, which may cause lethal complications, and sometimes death [4].

There is currently no specific antiviral therapy for dengue fever; once the disease is contracted, there is no way to combat it other than relying on the host’s immune response. Several vaccines are currently in development; however, given the current cost-effectiveness, efficacy, safety and estimated impact of vaccination, the WHO’s present recommendation is to introduce it only in geographic settings (national or sub national) where the disease is particularly problematic [5].

### Motivation for study

Climate change, specifically rising temperatures, is likely playing a crucial role in dengue transmission, potentially driving its expansion across the globe, as predicted by several studies [6–10]. Socio-economic conditions in a given location can be vital for a disease to persist once local transmission has occurred [11–15]; however, research in this domain, generally, does not account for socio-economic factors other than gross domestic product (GDP), which is a standard measure of the market value of all the final goods and services produced over a specific time period in a given location. Some studies have looked at the interaction between climate, socio-economic factors and demographics at a local level [16–20], focusing on factors specific to local areas, which means that their findings cannot be easily extrapolated to the macro level. To get better estimates of where dengue may spread, there is a need to understand how climate factors, socio-economic factors and demographic factors interact over a greater geographic scale to reveal common global patterns.

The original contribution of this article is that it selects factors shown to predict dengue at a local level and tests whether the association can be generalized to the regional or state level. In addition, we propose a more comprehensive set of socio-economic predictors of dengue transmission, to disentangle the role of GDP from other measures. Although a useful and parsimonious indicator, GDP is a very broad measure and it is not necessarily reflective of population health and well-being, distribution of wealth, discrimination and spending on public welfare [21]. More importantly, GDP alone may not be able to capture cross-regional differences. The predominance of using GDP as an indicator has been largely questioned [22–25]; for some time now researchers in human health geography, critical public health, and social epidemiology have requested more careful consideration of the contextual social and economic conditions that shape diseases at the local level [26, 27].

To this end, this study investigates regional differences in the incidence of dengue by evaluating the impact of socio-economic and demographic factors such as household income, regional rates of education, housing overcrowding, life expectancy, medical resources, migration flows, age structure of the population (the proportion of people under 14 and over 65), and population density.

The study focuses on the occurrence and distribution of dengue in Mexico and southern regions of the United States (US) where dengue has been reported, as some US regions share very similar environmental conditions but have distinct socio-economic conditions [12]. This study takes advantage of time series data between 2011 and 2019 and it is, therefore, able to exploit cross sectional variation between states, and variation over time for each state.

### Conceptual framework

Dengue transmission is determined by interactions between host, vector and pathogen, and modulated by ecological, climatic and geographic factors, including socio-economic factors. Regions were selected for the empirical analysis if conditions were met in terms of their *receptivity* and *vulnerability*, based on principles laid out in the WHO’s framework for malaria elimination [28].

Receptivity is defined as the ability of an ecosystem to allow transmission of a virus (dengue in this study). An ecosystem can be considered receptive if competent vectors, a suitable climate and a susceptible population are present; in other words, regions are selected if autochthonous virus transmission may occur because human populations and vector populations overlap/interact. Vulnerability occurs when either (1) a region was receptive and had regularly reported cases over the study period (endemic) or (2) bordered an endemic region and occasionally reported cases (likely due to spread or importation from neighbouring regions). We defined modulating factors as variables that influence the transmission dynamics of dengue such as host population size, host density, climate factors and medical interventions.

#### Receptivity

Since dengue is a vector borne disease, understanding the key ecological requirements of its vectors is crucial to assessing the receptivity of a region. As explained below, some of the main factors determining the receptivity of a region to dengue (due to the presence of its vectors) are: its physical environment (land use), the overlap with the human population, and its climate.

Both types of *Aedes* mosquitoes that transmit the dengue virus are ectothermic organisms and are highly sensitive to colder temperatures and extreme high temperatures. *A. albopictus* adults can survive in temperatures from 15 to 35 °C and *A. aegypti* from 10 to 35 °C [29], while their growth and development are severely inhibited in ambient temperatures below 13 °C or above 35 °C. *A. albopictus* eggs though, can go through diapause (suspended development) when exposed to extreme cold (down to − 10 °C). This adaptation allows them to inhabit environments with a wider annual temperature range, with more distinct seasonal changes than in tropical climates, where climate is more homogeneous. *A. aegypti* can endure a wider range of temperatures, but its survival at temperatures below 14–15 °C is limited to short periods, since its mobility is severely restricted and its ability to imbibe blood impeded. *A. aegypti* is also highly sensitive to fluctuations in temperatures. As for most mosquito species, availability of freshwater habitat, humidity and precipitation are highly indicative of their distribution in the environment.

To account for this, we selected a range of humidity and temperature variables for analysis which would capture mosquitoes’ living requirements.

#### Vulnerability

As direct measures of vulnerability we include spatial effects (neighbourhood structures) in our models in order to explicitly account for spill over effects with infected neighbouring regions (for a more detailed description see the methods section). Indirect measures of vulnerability can be derived from traditional patterns of travel and population flow in the area; indeed, well connected areas, in terms of trade and transport with considerable human movement, can benefit both mosquito species and dengue, by facilitating their movement and spread [30–32].

#### Modulating factors

Modulating factors can either speed up or slow down transmission. The transmission cycle of dengue is complex, since there are several key interactions at play between the virus, host and vector. Density of both the vector and host are fundamental factors in disease transmission, as contact between infected vectors and susceptible hosts is the source of new infections [33]. Mosquito breeding habitat can be increased by precipitation and flooding [34], temperature heavily influences mosquito hatching rate, development time [35, 36] and optimal temperature can shorted the extrinsic incubation period (EIP) [37]. While there are no datasets covering mosquito population abundance in all of our study regions, we selected meteorological variables that predict mosquito abundance and therefore are related to dengue transmission. Furthermore, there are several socio-economic risk factors of dengue including home water storage (rather than receiving piped water), poor sanitation, and poor public services (e.g. litter not collected) [12, 38–42]. Such factors can be responsible for creating breeding habitat for mosquitoes and bringing them into closer contact with humans, therefore increasing the risk of dengue. By contrast, use of mosquito nets, insect screens, and air-conditioning, can limit the chance of being bitten. Similarly, knowledge and education of mosquito ecology can also help people make personal interventions and reduce risk of being bitten [43]. Because there are no direct measures of home water storage or the use of mosquito nets, we use a range of socio-economic indicators as proxies, capturing a latent variable that would represent vector risk. The rationale is that people living in locations with better socio-economic conditions can avoid contact with mosquitoes and restrict virus transmission, either from the bottom-up (e.g. personal interventions) or the top-down (e.g. regional government pest control). However, it is important to note that factors associated with higher economic status can also bring humans into closer contact with mosquitoes, for example home owners with gardens, potted plants and ponds, or having good access to recreational space where mosquitoes can breed [44]. In terms of post-infection factors that influence dengue transmission, access to health care, risk perception and access to information on dengue infection symptoms had positive effects on people’s decision to seek medical help when presented with dengue infection symptoms [14, 43, 45]. To reflect this in the conceptual framework we selected variables that would proxy access to health care and variables which would represent access to information and personal knowledge. Finally, younger people are more likely to be infected by dengue [46], so we selected variables that represent the age structure of the population.

## Materials and methods

In this study, we compiled a spatial temporal data-set that would reflect the conceptual framework. By predicting the distribution of *A. albopictus* and *A. aegypti* in Mexico and the United States, we could determine which regions were receptive i.e. there was an overlap between the vector distribution and the human population at risk. By combing these results with reported cases of dengue, we could determine which regions were vulnerable. We then went on to collect data on modulating factors of dengue transmission in vulnerable regions. Furthermore, our vector distribution maps allowed us to extract more accurate data on the host population at risk and climatic factors that contribute to disease transmission.

### Species distribution models to estimate regional susceptibility

Because the exact distribution of vectors is unknown, we estimated the likelihood that a vector would occur in a region conditional on a set of covariates. More specifically, we estimated the distribution of the *Aedes* mosquitoes using a generalized additive logistic regression, with point location occurrence data as our dependent variable, and annual temperature range, mean temperature of the coldest quarter, precipitation during the driest quarter as covariates. Predictions were then used to select susceptible regions.

Point location occurrence data for *A. aegypti* and *A. albopictus* were obtained from a global geographic database of known occurrences between 1960 and 2014, compiled by members of the Institute of Biodiversity, Animal Health and Comparative Medicine, College of Medical, Veterinary and Life Sciences, University of Glasgow [47]. Point occurrence data represent spatial geo-coordinates of a location in which a given individual organism was sampled or sighted. Many of the samples in this data-set consists of museum records or unpublished studies including national entomological surveys. Since the data-set contained sparse information relating to the timings and frequency of each sample, we selected global observations from 1970 onward to capture the entire range of climatic conditions that each species can survive in, and to limit potential sample bias caused through the selection of localised seasonal collections. We also removed any duplicate observations i.e. replicate coordinates.

Climate data were extracted using R’s DISMO package in all point locations where mosquitoes occurred. Climate data for the species distribution prediction modelling were sourced from the MERRAclim, a database complied by members of the Department of Biology and Geology, Physics and Inorganic Chemistry, Rey Juan Carlos University [48]. This data-set was built using 2 m above surface air temperature (Kelvin degrees) and 2 m above surface specific humidity (kg of water/kg of air) satellite observations from NASA’s Modern Era Retrospective Analysis for Research and Applications Reanalysis.

**Fig. 1:**
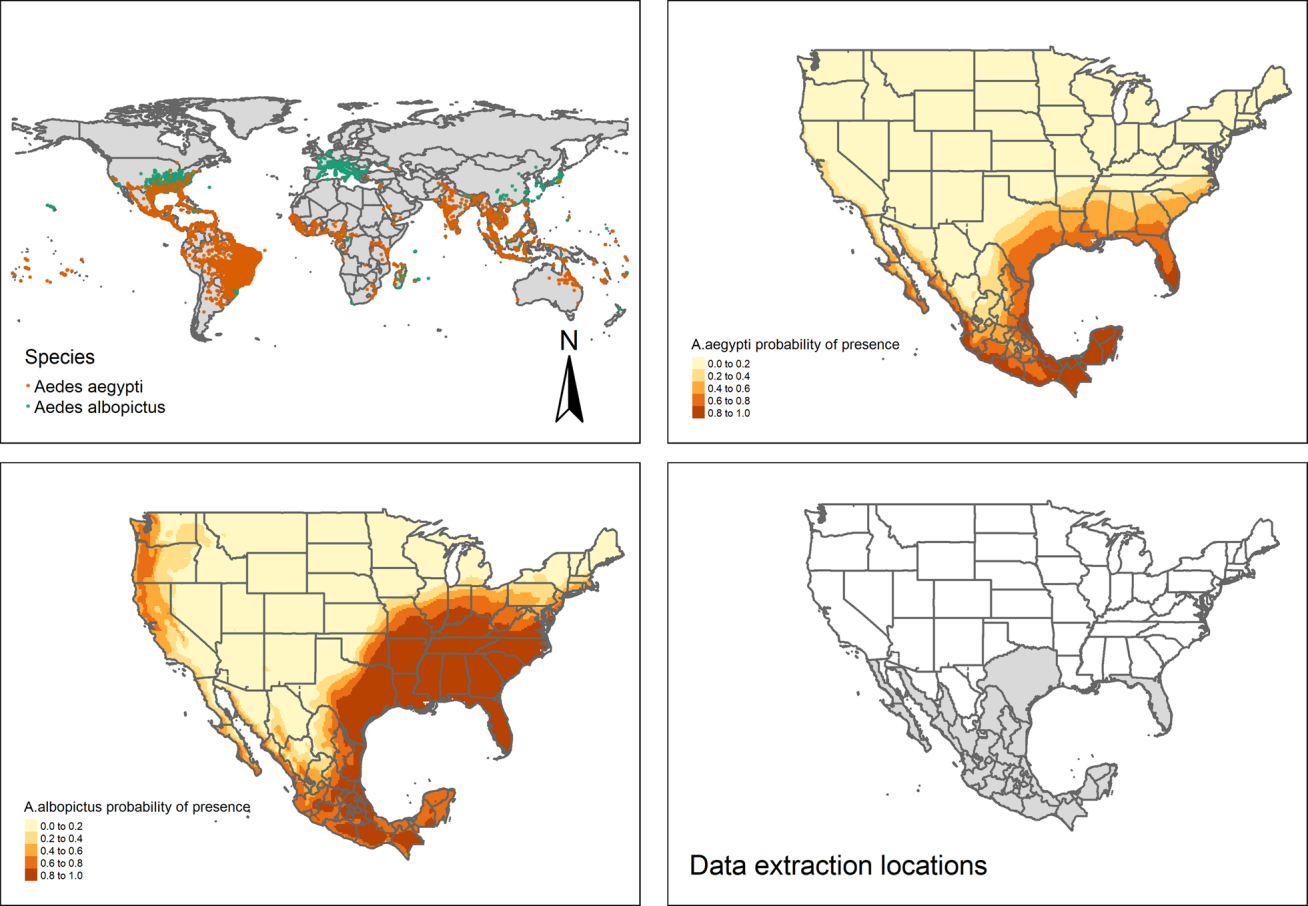
*Aedes* sample locations and SDM results 1 Top left: *Aedes* point locations. 2 Top right: Results of *Aedes aegypti* SDM 3 Bottom left: Results of *Aedes albopictus* SDM 4 Bottom right: Receptive regions/data extraction locations

Figure 1 shows the results of the modelling and *Aedes* sample locations. Tables providing summary statistics for the climate values at *Aedes* point locations can be found in Additional file 1. More specific information on statistical methods and results from this analysis can also found in Additional file 1.

### Data extraction and methods to assess the impact of climate, demographic and socio-economic factors on dengue

The Global Administrative Unit Layers [49] data-set along with our *Aedes* distribution maps (results Fig. 1, bottom right) were combined using R’s Sf package to create regional shape files that could spatially capture and process the human population and climate data for the main analysis. The GAUL data set contains geographic information in the form of shape files that lay out within country boundaries linked to a unique nomenclature. Countries are broken down into statistical subdivisions e.g., ADM0 representing data at country level (e.g. US), ADM1 at regional level (e.g. California).

Climate data for the main analysis i.e. measuring the impact of the climate variables on dengue transmission, were sourced from the Climate Prediction Center (CPC) of the National Centers for Environmental Prediction (NCEP), see [50]. These data represent a global summary of daily weather data. The CPC extracts surface synoptic weather observations from the Global Telecommunications System (GTS), which collects global data from a combination of weather station and satellite observations. Files were processed in R with the NetCDF, Raster and Dismo packages in order to create annual bio-climatic variables. The bio-climatic variables in this study were derived from daily maximum temperatures, daily minimum temperatures and total daily rainfall.

Population count data to predict the number of persons at risk in a region were sourced from the Socioeconomic Data and Applications Center’s Gridded Population of the World data set [51]. This data set estimates population count for the years 2000, 2005, 2010, 2015, and 2020, consistent with national censuses and population registers. Data were extracted from areas where vector presence was predicted. R’s Zoo package was used to replace values for missing years, by implementing a linear interpolation method that would predict trends between years. This way increases or decreases in human population were controlled for in the final model.

All spatial data was aggregated to the state level.

#### Dengue case data

Dengue case data for Mexico 2011–2019 were obtained from the Mexican Deputy General of Epidemiology web-page, which provided reports on all positive serious and non-serious cases of dengue (https://www.gob.mx). All data were provided at the regional level (ADM1 level). Case data for the United States were extracted from https://www.cdc.gov/arbonet, since data are provided at the county level (ADM2 level) we needed to aggregate them to the state level (ADM1 level) in order to match them with the main data-set.

#### OECD socio-economic and demographic data

Socio-economic and demographic data were extracted from the OECD’s Regional Statistics and Indicators Database [52]. This database provides comparable statistics and indicators and is presented in yearly time series. To capture factors determining the vulnerability of a region, we selected the variables “Inter-regional migration rate”, “Population density growth” and “Gross domestic product (GDP)”. For factors representing the socio-economic position of residents in a region we selected: “Household income”, “Life expectancy at birth”, and a measure of housing overcrowding “Number of rooms per person”. Furthermore, we selected “Secondary education” which would also help to capture areas where there is a higher proportion of manual labourer, e.g. agricultural workers or people working outdoors who may be more exposed to mosquitoes. We also selected “Perceived social network support”, “Self-evaluation of life satisfaction”, and “Perception of corruption” to try to capture additional features of a region, such as quality of life. Since these three variables yield some indication of how people perceive their surroundings and quality of life, we assume that poorer scores will capture poor infrastructure, poor public services, lack of basic provisions and lack of beneficial government intervention. To represent access to healthcare we selected “Active Physicians rate”, and variables which would represent access to information and personal knowledge i.e., “Broadband access” (however knowledge is also captured by “Secondary education”). Finally, younger people are more likely to be infected by dengue [47], so we selected variables that represent the age structure of the population i.e. “Percentage of Old Population Group (65+)” and “Percentage of Youth population group (0–14)”. Missing values were filled based on values for previous years or subsequent years, depending on their position in the data set.

All data were joined using the year of observation and region code, using R’s Dplyr package.

Table 1 provides summary statistics of all the collected data for the final models.

**Table 1:** Final dataset 2011–2019

Statistic	N	Min	Max	Mean	St. Dev.
Primary income of private households (USD per head)	306	2541.8	1,159,750.0	68,486.2	229,793.6
Regional Gross Domestic Product per capita	306	2883.4	1,044,310.0	64,153.2	206,614.7
Share of labour force with at least secondary education	306	26.9	89.5	42.1	13.0
Share of households with broadband access	306	7.3	85.2	41.5	18.9
Self-evaluation of life satisfaction	306	6	9	7.2	0.6
Perceived social network support	306	59	96	79.4	9.6
Perception of corruption	306	36.5	90.1	63.3	11.1
Active physicians rate (physicians for 1000 population)	306	0.7	4.8	1.6	0.6
Life expectancy at birth	306	70.5	79.4	75.1	1.4
Number of rooms per person	306	0.7	2.5	1.1	0.3
Inter-regional migration rate, (% migrants over population)	306	0.5	7.0	2.0	1.3
Population density growth	306	97.8	179.6	122.4	12.9
Percentage of Old Population Group (65+)	306	3.1	20.5	7.2	2.5
Percentage of Youth Population Group (0–14)	306	16.3	34.4	27.5	2.9
DGE Mexico confirmed serious dengue cases	306	0	5,041	259.3	606.0
DGE Mexico confirmed non-serious dengue cases	306	0	9,195	627.2	1,161.6
CDC US confirmed dengue cases	306	0	53	0.3	3.5
Population in aedes infected regions	306	335,728.3	28,145,145.0	4,710,557.0	5,484,386.0
Mean (C) temperature of warmest quarter	306	16.2	32.3	25.6	3.7
Mean (C) temperature of coldest quarter	306	9.0	25.3	18.0	4.3
Precipitation of driest quarter	306	0.0	8.3	1.1	1.3
Precipitation of warmest quarter	306	0.1	24.9	9.1	4.7

### Statistical methods

#### Factor analysis—data processing for regional analysis

A preliminary correlation analysis (see Additional file 1: Figures S8, S9- diagnostics) revealed how some of the socio-economic variables are strongly correlated with each other, and if included in a regression would give rise to multi-collinearity issues. By over-inflating the standard errors, multi-collinearity makes some variables statistically insignificant when they should be significant. To address this issue, following similar methods to [53], a factor analysis by maximum likelihood (VARIMAX rotation) was performed on socio-economic variables.

Factor analysis is a method for investigating whether a number of variables of interest $Y_1,Y_2,\ldots ,Y_n$, are linearly related to a smaller number of latent (i.e. ^∼^ not directly measured) factors $F_1,F_2,\ldots ,F_k$. The basic concept of factor analysis is that multiple observed variables have similar patterns because they are all associated with a latent variable. The factors are constructed in such a way that they capture the maximum amount of common variance (correlation) of the original items; the eigenvalue is a measure of how much of the variance of the observed variables a factor explains. The factor analysis can be formalized as follows:

$$\begin{aligned}Y_1=\beta _{10}+\beta _{11}F_1+\beta _{12}F_2+\cdots +\beta _{1k} F_k+\epsilon \\Y_2=\beta _{20}+\beta _{21}F_1+\beta _{22}F_2+\cdots +\beta _{2k}F_k+\epsilon \\Y_N=\beta _{n0}+\beta _{n1}F_1+\beta _{n2}F_2+\cdots +\beta _{nk}F_k+\epsilon \end{aligned}$$

Before performing the factor analysis, all variables had to be standardized to z-scores $(x-\mu )/\sigma$ to ensure that they were on the same scale. After performing the factor analysis, the predicted values for the factors for any individual region can be estimated. These predictions, known as factor scores, are weighted sums of the values of the observed items. Roughly, items with a stronger correlation with a factor component (i.e. those with larger loadings) will receive higher weights in the calculation of a score for that factor.

#### Quality of life index—data processing for regional analysis

We created a ‘Quality of Life Index’ by combining 3 variables from the OECD regional database: ‘Self-evaluation of life satisfaction’, ‘Perceived social network support’ and ‘Perception of corruption’. The variables were standardised, harmonised and combined into a composite indicator, capturing a latent quality of life measure, because each element on its own is unlikely to have a direct relationship with dengue.

#### General additive regression model to assess impact of independent variables on dengue case data at regional level

One of the main issues with our data-set is that it did not meet some basic assumptions for statistical inference, and specifically the data are not independent and identically distributed random variables (iid). More specifically, the data-set captured repeated measurements over the same regions, and observations were not independent because of spill over effects from neighbouring regions, therefore we needed to implement an appropriate statistical design to control for both temporal and spatial pseudo replication (lack of independence). We could deal with this in two ways, (1) either using a generalized linear mixed model (GLMM) approach, relaxing the assumption of independence and estimating the spatial/temporal correlation between residuals, or (2) model the spatial and temporal dependence in the systematic part of the model [54]. We opted to use a Generalized Additive Model (GAM) using R’s Mgcv statistical package because of its versatility and ability to fit complex models that would converge even with low numbers of observations and could capture potential complex non-linear relationships. One of the advantages of GAMs is that we do not need to determine the functional form of the relationship beforehand. In general, such models transform the mean response to an additive form so that additive components are smooth functions (e.g., splines) of the covariates, in which functions themselves are expressed as basis-function expansions. The spatial auto-correlation in the GAM model was approximated by a Markov random field (MRF) smoother, defined by the geographic areas and their neighbourhood structure. We used R’s Spdep package to create a queen neighbours list (adjacency matrix) based on regions with contiguous boundaries i.e. those sharing one or more boundary point. We used a medium rank MRF, which represented roughly one coefficient for two areas. The local Markov property assumes that a region is conditionally independent of all other regions unless regions share a boundary. This feature allowed us to model the correlation between geographical neighbours and smooth over contiguous spatial areas, summarising the trend of the response variable as a function of the predictors, for further information see section 5.4.2 of [55]. In order to account for variation in the response variable over time, not attributed to the other explanatory variables in our model, we used a saturated time effect for years, where a separate effect per time point is estimated.

We first tried to fit our model using a Poisson distribution. However, the mean of our dependent variable (dengue cases by region and year) was lower than its variance − E(Y) < Var(Y), suggesting that the data are over-dispersed. We also tried to fit our models using the negative binomial, quasi poisson and tweedie distribution, all particularly suited when the variance is much larger than the mean. After several tests, we concluded that the tweedie distribution worked well with our data and allowed us to model the incident rate. Analysis of model diagnostic tests didn’t reveal any major issues, in general residuals appeared to be randomly distributed (see Additional file 1: S10–S19—diagnostics).

Tweedie distributions are defined as subfamily of (reproductive) exponential dispersion models (ED), with a special mean-variance relationship.

A random variable $Y$ is Tweedie distributed $TW_{p}(\mu , \sigma ^2)$ if $Y \, ED(\mu , \sigma ^2)$, with mean = $\mu$ = $E(Y)$, positive dispersion parameter $\sigma ^2$ and $Var(Y) = \mu \sigma ^2$.

The empirical model can then be written as:

$$\begin{aligned} E(Y) = f_1(\text{X}_{it}) + f_n(\text{Year}_{t}) + f_m(\text{Region}_{i}) \end{aligned}$$

where the $f(.)$ stands for smooth functions; $E(Y)_{it}$ is equal to dengue incidence in region $i$ at time $t$, which we assume to be Tweedie distributed; $X{it}$—is a vector of socio-economic, demographic and climate variables. $Year_{t}$ is a function of the time intercept and $Region_{i}$ represents neighbourhood structure of region.

We run two separate sets of analyses: one comparing regions in the US and Mexico and another one looking at Mexico only, to check for robustness.

## Results

**Fig. 2:**
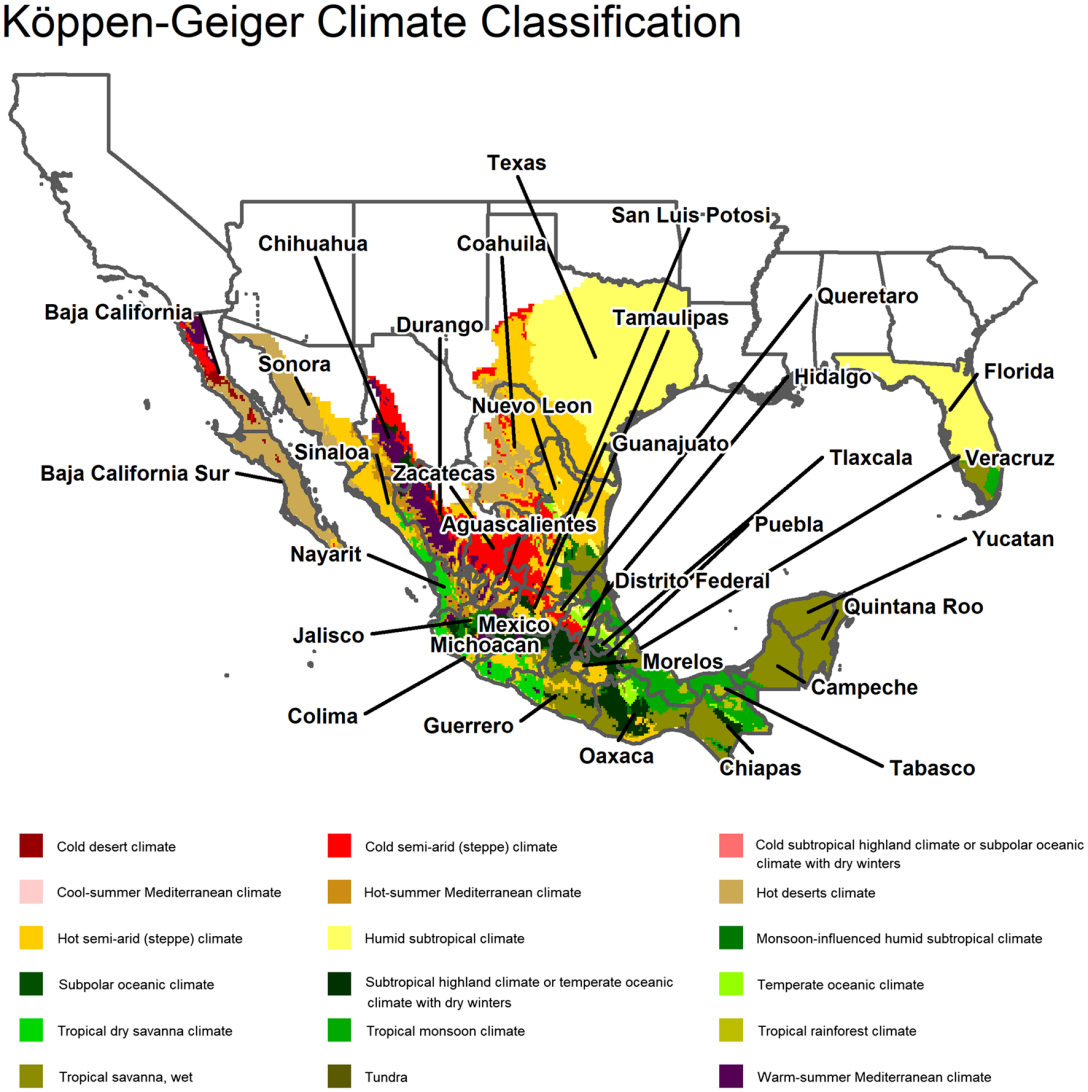
Koppen-Geiger climate classification in study regions (Source: koeppen-geiger.vu-wien.ac.at)

**Fig. 3:**
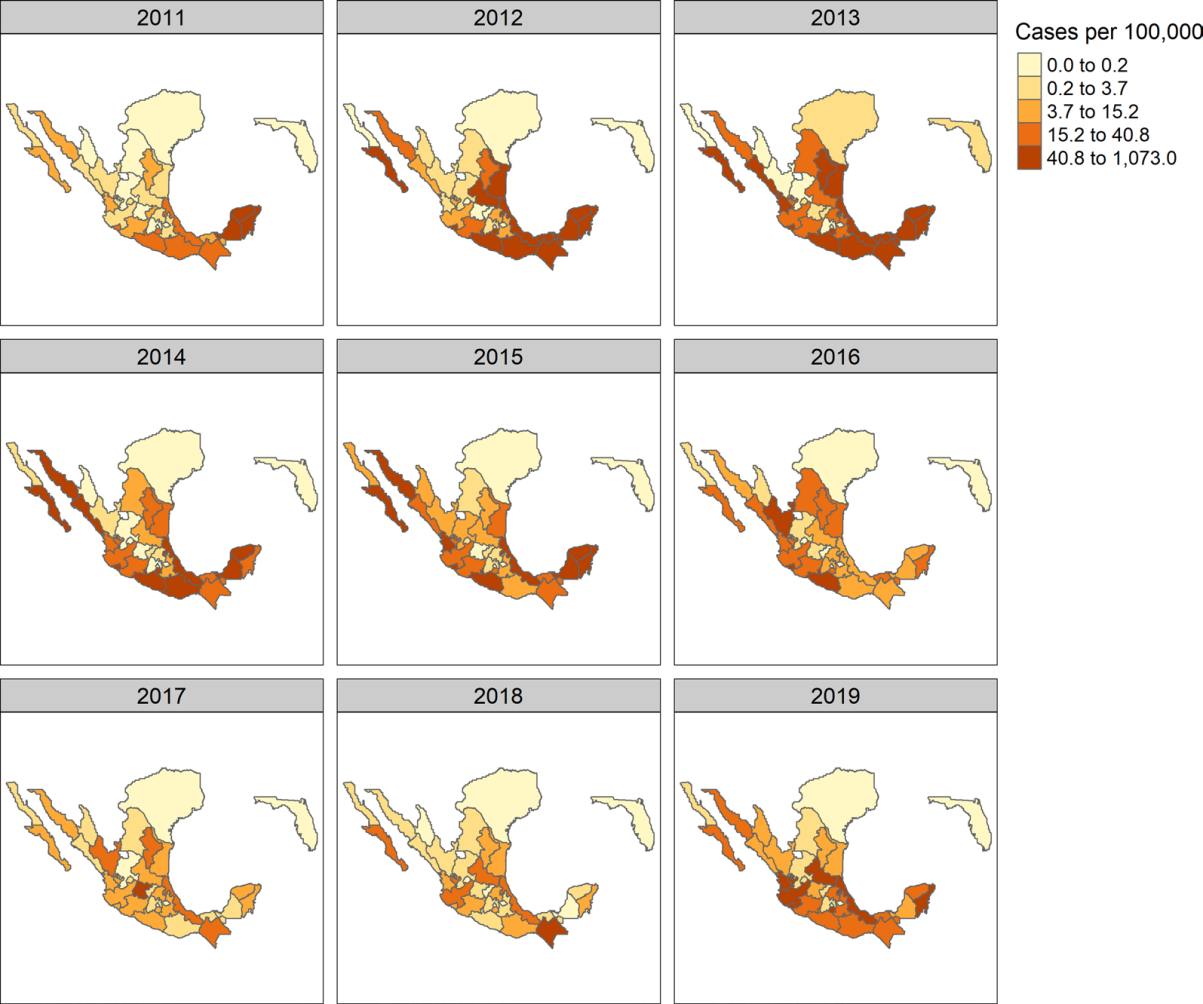
Crude incidence rates of dengue per 100,0000 people

Figures 2 and 3 provide a descriptive overview of the study regions, a characterisation of their environments and the reported disease incidence for those years. As we can observe, the majority of dengue cases are reported in tropical and sub tropical climates.

Tables 2 and 3 provide the results of the factor analysis i.e. the weighting of our socio-economic indicators. Table 4 shows the results for the regression model comparing confirmed dengue cases in the US and Mexico for 2011–2019. Table 5 restricts the analysis to Mexico only since we could exploit a better data-set in terms of case reporting, scale, and we could explore the impact of the socio-economic variables individually since, there was less correlation between Mexican regions.

**Table 2:** Socio-economic factor analysis results US/MEX

	Factor 1
Primary income of private households (USD per head)	0.91
Share of labour force with at least secondary education	0.95
Life expectancy at birth	0.78
Number of rooms per person	0.97

**Table 3:** Socio-economic factor analysis results Mexico

	Factor 1
Primary income of private households (USD per head)	0.41
Share of labour force with at least secondary education	0.95
Life expectancy at birth	0.59
Number of rooms per person	0.65

**Table 4:** Final regression models US/MEX: EDF value is reported as the coefficient, and DF is included in parentheses (not standard errors because a chi-square test is used for the smooth terms)

	GDP model	SE model	Dem model	Clim model	GDP full model	Full model
Intercept	3.19*** (0.26)	2.44*** (0.22)	2.53*** (0.23)	2.46*** (0.23)	2.59*** (0.22)	2.22*** (0.20)
Per capita GDP	0.00*** (0.00)				0.00*** (0.00)	
Socio-economic index		1.87*** (1.97)				1.78*** (1.92)
Active physicians per 1000		1.72** (1.90)			1.87*** (1.97)	1.88*** (1.98)
Households (%) with broadband access		1.94*** (1.99)			1.95*** (1.99)	1.95*** (1.99)
Quality of life index		1.78* (1.92)			1.00 (1.00)	1.00 (1.00)
Inter-regional migration rate			1.00*** (1.00)		1.65 (1.85)	1.61 (1.82)
Pop density growth			1.82 (1.96)		1.00*** (1.00)	1.00*** (1.01)
Percentage of population (65+)			1.97*** (1.99)		1.93*** (1.96)	1.79*** (1.89)
Mean temp (°C) of coldest quarter				1.90*** (1.98)	1.88*** (1.97)	1.83*** (1.95)
Precip of warmest quarter (in)				1.57* (1.81)	1.63* (1.84)	1.53** (1.76)
Year	7.27*** (8.00)	6.75*** (8.00)	6.99*** (8.00)	7.28*** (8.00)	6.61*** (8.00)	6.61*** (8.00)
Region	13.3*** (13.91)	12.76*** (13.72)	13.04*** (13.69)	12.72*** (13.81)	10.46*** (12.24)	10.45*** (12.19)
AIC	2339.82	2322.32	2332.83	2285.47	2233.10	2231.57
Deviance	1288.59	1205.43	1257.24	1143.83	967.55	962.59
Deviance explained	0.51	0.55	0.53	0.59	0.66	0.66
Dispersion	3.52	3.37	3.47	3.20	2.84	2.83
$\text {R}^2$	0.36	0.42	0.40	0.40	0.51	0.51
Num. obs.	306	306	306	306	306	306
Num. smooth terms	2	6	5	4	10	11

*** p < 0.01; ** p < 0.05; * p < 0.1

**Table 5:** Final regression models Mexico: EDF value is reported as the coefficient, and DF is included in parentheses (not standard errors because a chi-square test is used for the smooth terms)

	SE model	Dem model	Clim model	GDP full model	SEindex model	Full model
Intercept	2.57*** (0.20)	2.89*** (0.21)	2.71*** (0.23)	2.20*** (0.24)	2.46*** (0.19)	2.44*** (0.18)
Per capita GDP				0.00* (0.00)		
Socio-economic index					1.96*** (1.99)	
Income of private households	1.95*** (2.00)					1.83** (1.96)
Share households with broadband	1.88*** (1.98)			1.93*** (1.99)	1.93*** (1.99)	1.92*** (1.99) (1.99)
Active physicians (1000 pop)	1.00*** (1.00)			1.81*** (1.95)	1.75*** (1.92)	1.81*** (1.95)
Number of rooms pp	1.95*** (1.99)			1.00 (1.00)		1.00 (1.00)
Labour force with secondary edu	1.97*** (2.00)			1.95*** (1.99)		1.95*** (1.99)
Quality of Life index	1.00 (1.00)			1.00 (1.00)	1.00 (1.00)	1.00 (1.00)
Inter-regional migration rate		1.00*** (1.00)		1.00 (1.00)	1.00 (1.00)	1.00* (1.00)
Pop density growth		1.51 (1.74)		1.00* (1.00)	1.00 (1.00)	1.00* (1.00)
Mean temp (°C) of coldest quarter			1.83*** (1.96)	1.89*** (1.98)	1.94*** (1.99)	1.89*** (1.98)
Precip of warmest quarter (in)			1.53 (1.76)	1.71* (1.90)	1.68 (1.88)	1.71* (1.90)
Year	6.72*** (8.00)	6.90*** (8.00)	7.27*** (8.00)	6.45*** (8.00)	6.53*** (8.00)	6.44*** (8.00)
Region	13.18***(13.89)	13.17***(13.90)	12.07***(13.57)	12.19***(13.52)	12.32*** (13.59)	12.13*** (13.47)
AIC	2251.33	2340.28	2270.07	2207.77	2212.08	2204.83
Deviance	978.13	1242.10	1071.46	871.99	886.18	859.68
Deviance explained	0.61	0.47	0.57	0.66	0.66	0.67
Dispersion	3.00	3.57	3.17	2.76	2.78	2.73
$\text{R}^2$	0.47	0.38	0.40	0.52	0.51	0.51
Num. obs.	288	288	288	288	288	288
Num. smooth terms	8	4	4	11	10	12

*** p < 0.01; ** p < 0.05; * p < 0.1

### US/Mex analysis

#### Socio-economic and demographic indices Mexico/US

It was not possible to explore the individual impact of all of the variables in our data-set because of collinearity issues. Population density was found to be positively correlated with GDP and primary income. “Percentage of Old Population Group (65+)” was negatively correlated with “Percentage of Youth Population Group (0–14)” (see Additional file 1: S8, S9) diagnostics). For this reason, we performed a factor analysis to reduce the number of variables, as explained in more detail in the section on statistical methods. The Mexico/US factor analysis captured the variance in 4 highly correlated variables: higher share of labour force with at least secondary education, more rooms per inhabitant, life expectancy at birth, primary income of households, and yielded one composite indicator (see Table 2) , which we included as a regressor. A priori, the socio-economic indicator is expected to have a negative association with dengue.

We built our statistical model in a stepwise fashion so we could analyse it using the lowest Akaike Information Criterion (AIC) which would help us validate the quality of statistical models for our dataset. The first column of Table 4 (GDP Model) shows the association between regional GDP and dengue cases across the regions; the second column (SE Model) shows the association between regional dengue cases and the socio-economic indicator derived through factor analysis, plus other variables such as active physician rate, broadband access and the quality of life index. Column 3 (Dem Model) includes demographic variables, such as inter-regional migration rate, population density growth and the percentage of older population (65+). Column 4 (Clim Model) includes the climate variables mean temperature of the coldest quarter and precipitation in the warmest quarter. The “full model” in column 5 shows the relationship between dengue incidence and all explanatory variables in our final model. Table 4 also summarises the relevant statistics (AIC, Deviance, Adjusted R squared and so on) to compare the different specifications; the full model has the best fit (lower AIC and higher adjusted R squared), followed by the one in which we control only for the climate variables (as well as the year, regional effects); the first model, controlling for GDP alone, has the highest AIC and has a worse fit than the specification including the socio-economic indicators.

When controlling for demographic and climate variables, the impact of the socio-economic indicators still remains statistically significant, as well as the impact of temperature.

**Fig. 4:**
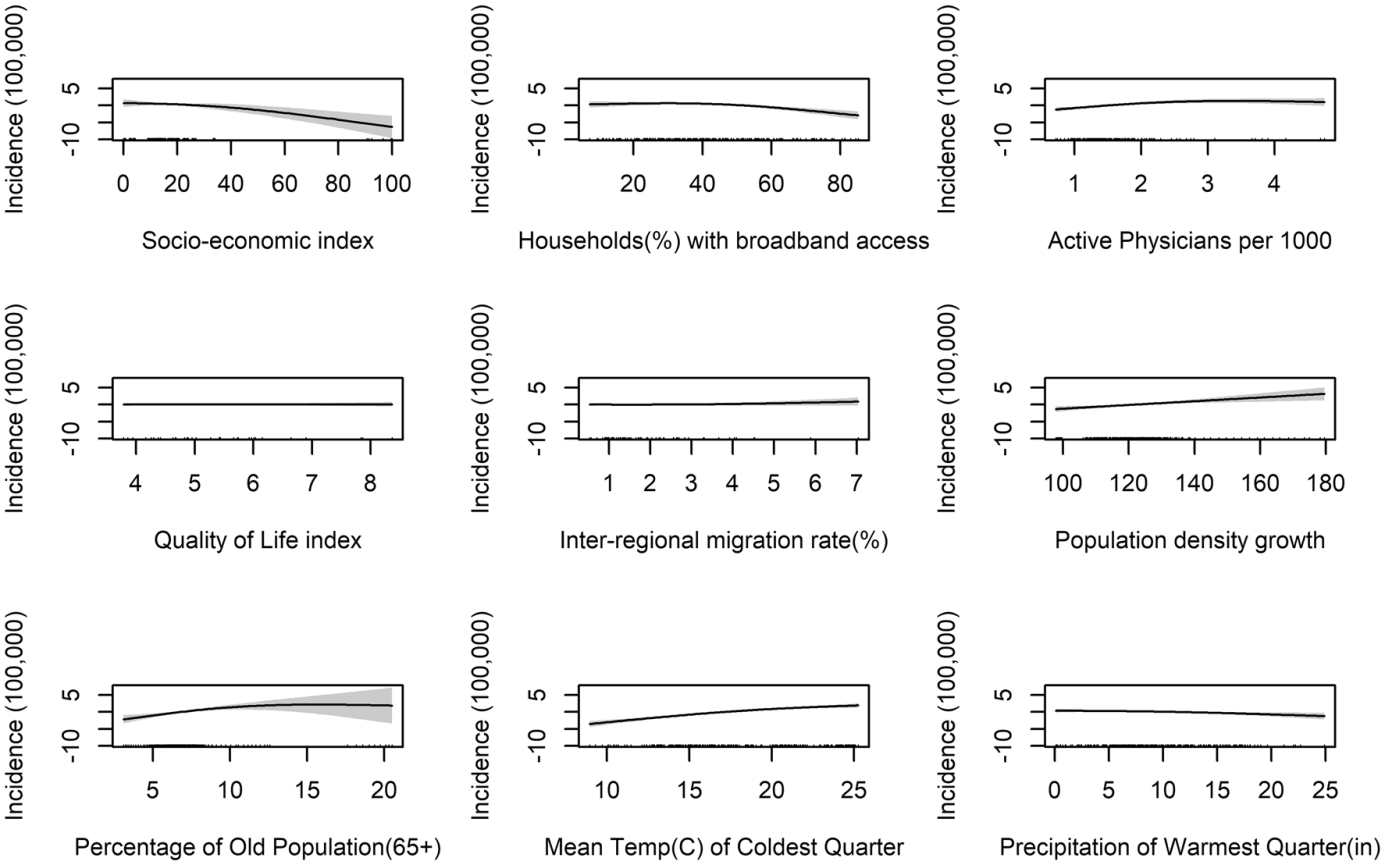
Partial effects of explanatory variables: GAM Mex/US model

Please note that as we are not estimating a standard regression model, the figures reported should not be read as coefficients, but degrees of freedom of the smooth terms. Given that we cannot interpret the coefficients to infer the sign and magnitude of the relationship, we visualise it by plot. Figure 4 plots the partial effects—the relationship between a change in each of the covariates and a change in the fitted values in the full model; the first plot shows that the socio-economic index has linear negative impact, but the relationship becomes weaker at very high scores; given the weight of each variable in the factor analysis, the results can be interpreted as an increase in the share of labour force with at least secondary education, more rooms per inhabitant, life expectancy at birth, primary income of households are associated with fewer dengue cases. Regions with better broadband access tend to be those with lower incidence rates of dengue, however in this case the relationship is flat at low levels of broadband coverage (below 40 percent) and then turns negative and quadratic at higher levels of access. These results could suggest residents are more likely to search for information on dengue prevention measures consequently lowering transmission potential, or when suffering with symptoms may be more likely to seek medical advice, therefore breaking the transmission cycle; these results are consistent with findings by [56, 57]. This result also could be an indicator of more advance and urbanized regions vs agricultural and less developed regions. It is reported that dengue tends to affect more those working in labour-intensive industries, such as agriculture or fishing [58, 59].

The variable representing active physician rates has a positive impact on the incidence of dengue, in that regions with more active physicians tend to have higher incidence; however, this is likely due to more accurate reporting. Even in this case, the relationship is concave—positive up to 3 percent rate and flat afterwards.

The impact of the demographic variables on the incidence of dengue also follows the expected sign, with inter-regional migration rate and population density growth being associated with a linear increase in the incidence of dengue; the presence of an older population is associated with higher incidence of dengue up to a certain level—it peaks at around 14 percent—and then a reduction, as can be seen from Fig. 4. One possible explanation for this is that a higher proportion of older people means a more vulnerable population, however very high rates are also associated with wealthier regions, which offset the main impact of age. Figure 4 also show the impact of the Mean temperature (°C) of coldest quarter variable is almost linear. We can see that most cases occur in regions which have particularly mild cold seasons. This is concurrent with the literature, we would expect to see more cases of dengue in regions with tropical climates, where there is a distinct absence of a cold season, during which low temperatures would kill the mosquitoes off or cause mosquitoes to overwinter effectively inhibiting disease transmission, instead such conditions allow the virus and mosquitoes to persist throughout the year.

The relationship between rainfall and dengue incidence in the full model is slightly negative and significant; even though this finding could appear counter intuitive, it is probably due to the fact that mosquito larvae can be washed away during intense rainfall [60]. Furthermore, both *Aedes* mosquitoes can survive in drier climates than expected, by exploiting artificial water sources and man-made habitats, as already mentioned “Introduction” section.

### Mex analysis

**Fig. 5:**
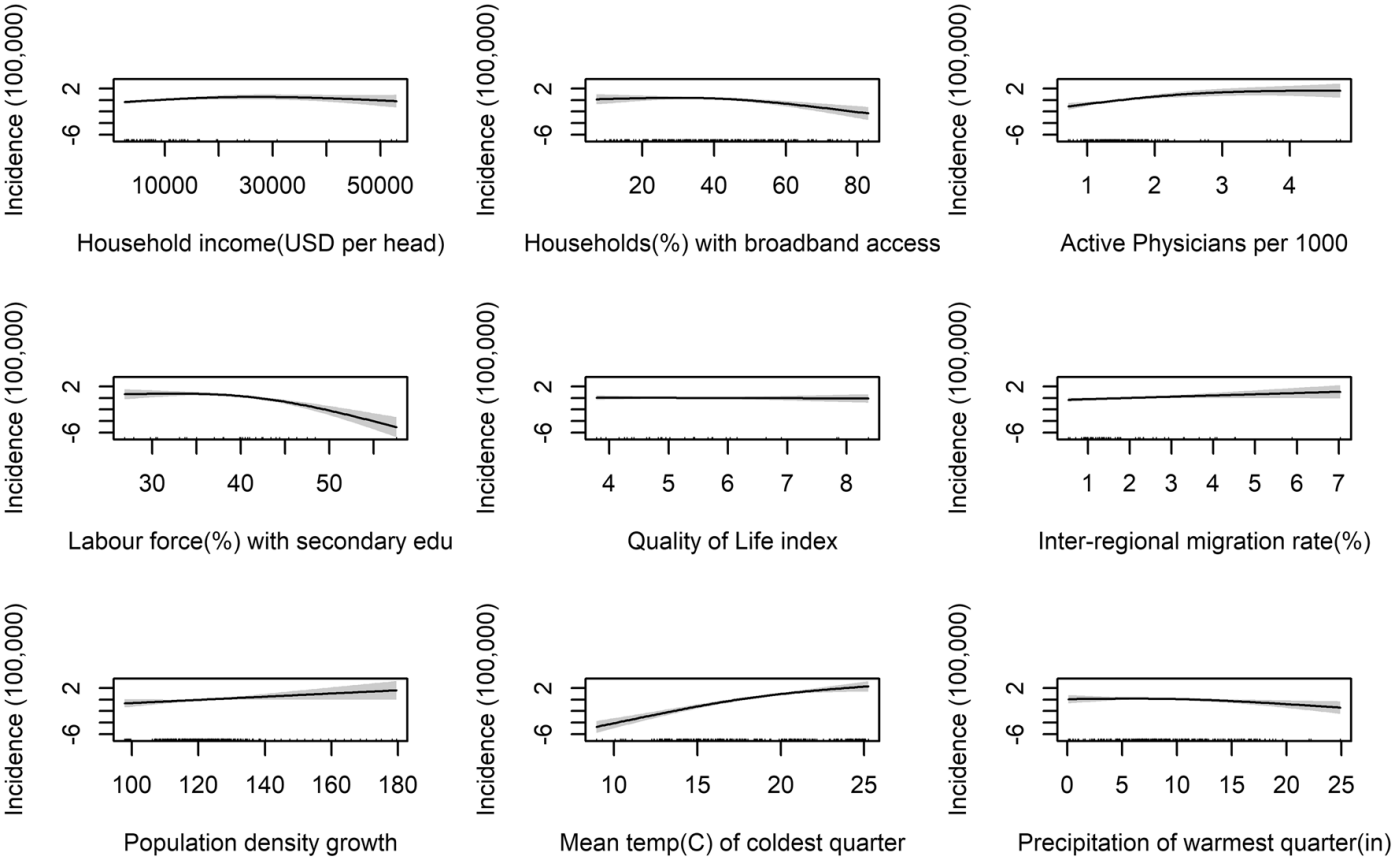
Partial effects of explanatory variables: GAM Mexico model

For our second analysis focusing on differential diffusion of dengue within Mexican regions, we were able to analyse variables individually since there there is significantly less correlation between the socio-economic variables. However, we could not select ”Population density” because of a correlation with ”Primary income of household”s and ”GDP”. ”Percentage of old population Group (65+)” was negatively correlated with ”Population density growth” so was not included in the final model. Furthermore, ”Percentage of population share (0–14)” was highly correlated with ”Access to broadband” and ”Workforce with secondary education” (and negatively correlated with population 65+), so we didn’t include it in the study. We again built our second statistical model in a stepwise fashion so we could analyse it using the lowest Akaike Information Criterion (AIC) which would help us validate the quality of statistical models for our data set. Figure 5 and Table 5 present the results of our second analysis focusing only on Mexican regions.

Our findings for the second analysis are similar to the first: the most significant variables are “Share of households with internet access”, “Active Physicians Rate (1000 pop)” and “Mean temperature (C) of coldest quarter”. Our socio-economic indicator was a good predictor of dengue incidence, although when “GDP” was paired with other individual variables from the factor analysis (except primary income) it helped to create a very useful model. The best fit model was our final specification using our socio-economic variables individually; however, primary income of households is not a reliable predictor of dengue, since, by the concave relationship, it would appear that gains in economic activity may increase the spread of the virus (for instance because of movement of goods and people), but could also be correlated with higher reporting. One of the strongest predictors of dengue in our final specification is “Share of labour force with secondary education”. As previously noted, this is consistent with other findings by [58, 59] as dengue tends to affect more those working in labour-intensive industries, such as agriculture or fishing.

## Discussion and conclusions

The study investigated the impact of socio-economic, demographic and climate variables on the distribution of dengue. Its original contribution is that it selected factors shown to predict dengue at a local level and tested whether the association could be generalized to the regional or state level. In addition, it showed the potential development of more sophisticated socio-economic indicators using regional and internationally available data. The study identified which regions are most at risk, by estimating where dengue vectors are likely to occur given their suitability to climate conditions in terms of receptivity and vulnerability. By estimating the chance of a vector occurring in a region, we could then assess the impact of socio-economic, demographic and climate factors on the incidence of dengue. The results confirmed a strong association between our novel indices of socio-economic factors and dengue cases per region. Such results are consistent with the findings reported by [12, 14, 39–42, 45, 61]. Two main lessons can be drawn from this study: first, while higher GDP is generally associated with a drop in the incidence of dengue, a more granular analysis revealed that the crucial factors are a rise in education (with fewer jobs in the primary sector) and better access to information or technological infrastructure. For this reason, the use of more sophisticated measures, aside from GDP, should be taken into account when building models that try to predict disease distribution. The use of more granular socio-economic indicators can explain with greater accuracy the differences in the spread of disease in places with similar physical geography and ecological characteristics. In addition, public health authorities should be aware of the presence of non-linearities in relationships between dengue and income. Secondly, factors that were shown to have an impact of dengue at the local level are also good predictors at the regional level. Given that data for these indicators are available at a sub-national scale for OECD countries and selected OECD non-member economies, these indices may help us better understand factors responsible for the global distribution of dengue and also, given a warming climate, may help us to better predict vulnerable populations. Although the variables used in this study do not represent disease transmission mechanisms directly, understanding the relative impact of socio-economic, demographic and climate factors on disease outcomes can help risk assessors predict where diseases are likely to occur in the future, by identifying locations with vulnerabilities in public health systems and/or by identifying impoverished areas that tend to be susceptible to disease. Our findings are not only useful for public health, but also contribute to a wider scholarly debate on whether and to what extent can economic growth (measured via GDP) contribute to better outcomes of health and well-being. Finally, it is important to note that, with any analysis dealing with regional data, results should be taken with caution because of issues of scale and uncertainty introduced by the aggregation procedure. Further studies seeking to test the robustness of the indicators examined in this study should try to source data at a more refined scale, and test how these indicators can generalise across the different scales.

## Supplementary information

**Additional file 1.** Species distribution methods and results to estimate receptivity + model diagnostics (all models).

## Data Availability

The R project folder, main spatial dataset and R code for the project is available from https://doi.org/10.5281/zenodo.887909.

## Abbreviations

GDP: Gross Domestic Product
Mex: Mexico
US: United States of America
SDM: Species distribution model
ADM: Administrative division
