# Supplementary material for: Influence of socio-economic, demographic and climate factors on the regional distribution of dengue in the United States and Mexico
Source: Int J Health Geogr. 2020 Nov 2;19:44. doi: 10.1186/s12942-020-00241-1 (PMC7607660; doi:10.1186/s12942-020-00241-1)
Supplement: Supplementary file 1 — Additional file 1. Species distribution methods and results to estimate receptivity + model diagnostics (all models). [file 12942_2020_241_MOESM1_ESM.zip › 12942_2020_241_MOESM1_ESM.pdf]

Additional Files
S1
4.7.1 Vector and Environmental Relationships: Regression Analysis (Step 1)
To assess the relationships between vector and environment, techniques were adapted from [58]. Since our species distribution data set only consisted of presence data, absence data was substituted with background data. Background data points were sampled randomly from the study area, matching roughly the number of observations in the presence data. (Mex/US). Using background data allows us to characterize environments in the study region, which establishes the environmental domain of the study, whilst presence data should represent the conditions a species is more likely to be present than on average. Since vector presence is indicated by a binary variable equal to 1 and absence equal to 0, a relationship between environmental and vector presence was estimated with a logit model by maximum likelihood. Logit estimation techniques are based on the assumption that there is a latent variable y and that this latent variable is a linear function of all the explanatory variables. Climate data for the species distribution prediction modelling were sourced from MERRAclim [52]. This data-set was built using 2m air temperature (Kelvin degrees) and 2 m specific humidity (kg of water/kg of air) hourly data derived from satellite observations from the Modern Era Retrospective Analysis for Research and Applications Reanalysis. Tables S2 and S3 provide summary statistics for these data sets.
In order to predict if a vector was present in a given location i, the following equations were used

$$Pr(Aedes.aegypti = 1) = \beta_1 Tvar1_i + \beta_2 Tvar2_i + \beta_3 Pvar1_i + \epsilon$$

$$Pr(Aedes.albopictus = 1) = \beta_1 Tvar1_i + \beta_2 Tvar2_i + \beta_3 Pvar1_i + \epsilon$$

where X_i is a vector of regressors/independent variables (in this case: Tvar 1 represents temperature annual range; Tvar2 represents mean temperature of the coldest quarter; Pvar1 represents precipitation of the driest month and $epsilon$ is the error term.

4.7.2 Vector and Environmental Relationships: regression analysis results
The impact of climate on the probability of a vector being present was assessed by running a GAM logit regression. Results are reported in Table S4 and model diagnostics in additional files S6-S7.
All three predictors were highly significant for both *A. aegypti* and *A. albopictus*. The results from the GAM logistic regressions the study confirmed a positive and highly significant association between some climatic factors and vector presence; results were consistent with previous studies [28,45,55]:
A. albopictus seems to be distributed in environments that are warmer and wetter during the hotter months, *A. aegypti* seems to be sensitive to colder temperatures and temperature range.
For both species minimum temperature is a major limiting factor affecting distribution and this is conclusive with our results.
The models were validated using an assessment of the residuals and using a 5-fold cross-validation of the operating characteristic (ROC) curve, which compared false and true positives (see S6-S7). The data set was divided into 5 (k-5) subsets, and the model run 5 times. For each run, one of the k subsets is used as the test the rate of false positives and true positives. Both models demonstrated high accuracy in terms of predicting true positives with values not falling under 0.87 for *A. aegypti* and 0.89 for *A. albopictus*.

Table S2 A. aegypti climate variable summary

Statistic	N	Min	Max	Mean	St. Dev.
Temp.Annual.Range	8,584	4.600	55.200	23.880	8.032
Mean.Temp.Coldest.Quarter	8,584	−7.600	30.700	23.470	3.438
Precip.of.Driest.Month	8,584	348	2,493	1,488.795	321.253

Table S3 A. albopictus climate variable summary

Statistic	N	Min	Max	Mean	St. Dev.
Temp.Annual.Range	7,425	46	588	289.262	96.656
Mean.Temp.Coldest.Quarter	7,425	−83	307	191.324	65.688
Precip.of.Driest.Month	7,425	267	2,493	1,326.908	317.151

	A.aegypti	A.albopictus
Intercept	-2.14^{***}	-6.56^{***}
	(0.51)	(0.67)
Temperature annual range	2.04^{**}	2.78^{***}
	(2.26)	(2.96)
Mean temperature of coldest quarter	2.12^{***}	2.99^{***}
	(2.32)	(3.00)
Precipitation of driest month	2.83^{***}	2.98^{***}
	(2.97)	(3.00)
AIC	13478.91	11510.16
BIC	13541.08	11584.66
Log Likelihood	−6731.47	−5745.33
Deviance	13462.94	11490.65
Deviance explained	0.45	0.46
Dispersion	1.00	1.00
R^2	0.52	0.52
GCV score	−0.24	−0.25
Num. obs.	17798	15326
Num. smooth terms	3	3

$^{***}p < 0.001$; $^{**}p < 0.01$; $^{*}p < 0.05$

Table S4 Aedes GAM SDM results

	NB US/Mex	TW US/Mex	QP US/Mex	NB Mex	TW Mex	QP Mex
(Intercept)	-9.44^{***}	2.22^{***}	-9.12^{***}	-9.20^{***}	2.44^{***}	-8.75^{***}
	(0.26)	(0.20)	(0.26)	(0.18)	(0.18)	(0.21)
EDF: s(socio_economic_index_norm1)	1.65^{***}	1.78^{**}	1.92			
	(1.82)	(1.92)	(1.97)			
EDF: s(BB_ACC)	1.95^{***}	1.95^{***}	1.88^{**}	1.92^{***}	1.92^{***}	1.80
	(1.99)	(1.99)	(1.97)	(1.99)	(1.99)	(1.94)
EDF: s(DOC_RA)	1.68^{*}	1.88^{***}	1.95^{***}	1.34^{**}	1.81^{***}	1.95^{***}
	(1.88)	(1.98)	(1.99)	(1.54)	(1.95)	(1.99)
EDF: s(FLOWMOB_ALL_RA)	1.00	1.61	1.57	1.00^{*}	1.00	1.00
	(1.00)	(1.82)	(1.77)	(1.00)	(1.00)	(1.00)
EDF: s(QOL_index)	1.00	1.00	1.00	1.00	1.00	1.00
	(1.00)	(1.00)	(1.00)	(1.00)	(1.00)	(1.00)
EDF: s(POP_DEN_GR)	1.84^{***}	1.00^{**}	1.28^{*}	1.00	1.00	1.01
	(1.95)	(1.01)	(1.44)	(1.00)	(1.00)	(1.02)
EDF: s(Y65_MAX)	1.92^{***}	1.79^{**}	1.15^{**}			
	(1.96)	(1.89)	(1.24)			
EDF: s(bio11)	1.77^{***}	1.83^{***}	1.68^{***}	1.87^{***}	1.89^{***}	1.00^{***}
	(1.92)	(1.95)	(1.86)	(1.97)	(1.98)	(1.00)
EDF: s(bio18)	1.65^{*}	1.53^{*}	1.00	1.86^{*}	1.71	1.00
	(1.86)	(1.76)	(1.00)	(1.97)	(1.90)	(1.00)
EDF: s(fyear)	6.79^{***}	6.61^{***}	6.67^{***}	$\mathbf{5.97}^{***}$	6.44^{***}	6.46^{***}
	(8.00)	(8.00)	(8.00)	(8.00)	(8.00)	(8.00)
EDF: s(factor(ADM1_CODE))	11.54^{***}	10.45^{***}	9.30^{***}	12.35^{***}	12.13^{***}	10.32^{***}
	(12.97)	(12.19)	(11.22)	(13.56)	(13.47)	(12.31)
EDF: s(INCOME_PRIM)				1.84^{*}	1.83^{*}	1.93^{**}
				(1.96)	(1.96)	(1.99)
EDF: s(ROOMS_PC)				1.00	1.00	1.00
				(1.00)	(1.00)	(1.00)
EDF: s(EDU38_SH)				1.97^{***}	1.95^{***}	1.78
				(2.00)	(1.99)	(1.92)
AIC	3989.14	2231.57	–	3866.02	2204.83	–
BIC	4130.53	2373.21	–	4006.05	2350.39	–
Log Likelihood	−1956.60	−1077.75	–	−1894.78	−1062.68	–
Deviance	332.45	962.59	179965.91	303.55	859.68	169568.80
Deviance explained	0.61	0.66	0.75	0.62	0.67	0.70
Dispersion	1.00	2.83	881.70	1.00	2.73	957.09
R^2	0.09	0.51	0.57	0.09	0.51	0.58
GCV score	2011.57	1127.23	1180.27	1950.50	1119.74	1117.09
Num. obs.	306	306	306	288	288	288
Num. smooth terms	11	11	11	12	12	12

$^{***}p < 0.001$; $^{**}p < 0.01$; $^{*}p < 0.05$

Table S5 Model diagnostics - distributions

Figure S6 ROC + residual check A.aegypti model

Figure S7 ROC + residual check A.albopictus model

Figure S8 Correlation matrix: US/Mex dataset

Figure S9 Correlation matrix: US/Mex dataset

Figure S10 Diagnostics: Mex/US main model

Figure S11 Diagnostics (cont): Mex/US main model

Figure S12 Plotted residuals: Mex/US main model

Figure S13 Diagnostics: Mex main model

Figure S14 Diagnostics(cont): Mex main model

Figure S15 Diagnostics: Plotted residuals - Mex main model

Figure S16 Diagnostics - Negbin Mex/US main model

Figure S17 Diagnostics: Mex main model

Figure S18 Diagnostics: Quasi-poisson Mex/US main model

Figure S19 Diagnostics: Quasi-poisson Mex main model

	VAR	Unit	Indicator	Source
1	bio11	degrees C	Mean_Temperature_of_Coldest_Quarter	Climate Prediction Center
2	bio18	Precip mm	Precipitation_of_Warmest_Quarter	Climate Prediction Center
3	FLOWMIG_ALL_RA	Ratio	Inter-regional migration rate, (% migrants over population)	OECD regional database
4	GDP	USD per head	Regional GDP	OECD regional database
5	POP_DEN_GR	Index	Population density growth index (2001=100)	OECD regional database
6	ROOMS_PC		Average number of rooms per inhabitant (rooms per capita)	OECD regional database
7	Y0_14	Persons	Youth Population Group (0-14)	OECD regional database
8	Y65_MAX	Persons	Old Population Group (65+)	OECD regional database
9	DOC_RA		Active Physicians Rate (physicians for 1000 population)	OECD regional database
10	LIFE_EXP	Years	Life Expectancy at Birth	OECD regional database
11	INCOME_PRIM	USD per head	Primary Income of Private Households	OECD regional database
12	BB_ACC	Percentage	Share of households with internet broadband access	OECD regional database
13	EDU38_SH	Percentage	Share of labour force with at least secondary education	OECD regional database
14	INCOME_DISP	US Dollar	Disposable income per capita	OECD regional database
15	ROOMS_PC	Ratio	Number of rooms per person	OECD regional database
16	SUBJ_LIFE_SAT	Index	Self-evaluation of life satisfaction	OECD regional database
17	SUBJ_PERC_CORR	Percentage	Perception of corrution	OECD regional database
18	SUBJ_SOC_SUPP	Percentage	Perceived social network support	OECD regional database
19	dengue_non_serious	cases	number of non-serious cases reported in mexico	www.gob.mex
20	dengue_serious	cases	number of non-serious cases reported in mexico	www.gob.mex
21	dengue_cdc	cases	number of cases reported in USA	www.cdc.gov/arbonet
22	pop	population	human population in aedes infected areas	https://sedac.ciesin.columbia.edu

Table S20 Main analysis variable description and codes
