# Supplementary figures and images for: Influence of socio-economic, demographic and climate factors on the regional distribution of dengue in the United States and Mexico
Source: Int J Health Geogr. 2020 Nov 2;19:44. doi: 10.1186/s12942-020-00241-1 (PMC7607660; doi:10.1186/s12942-020-00241-1)

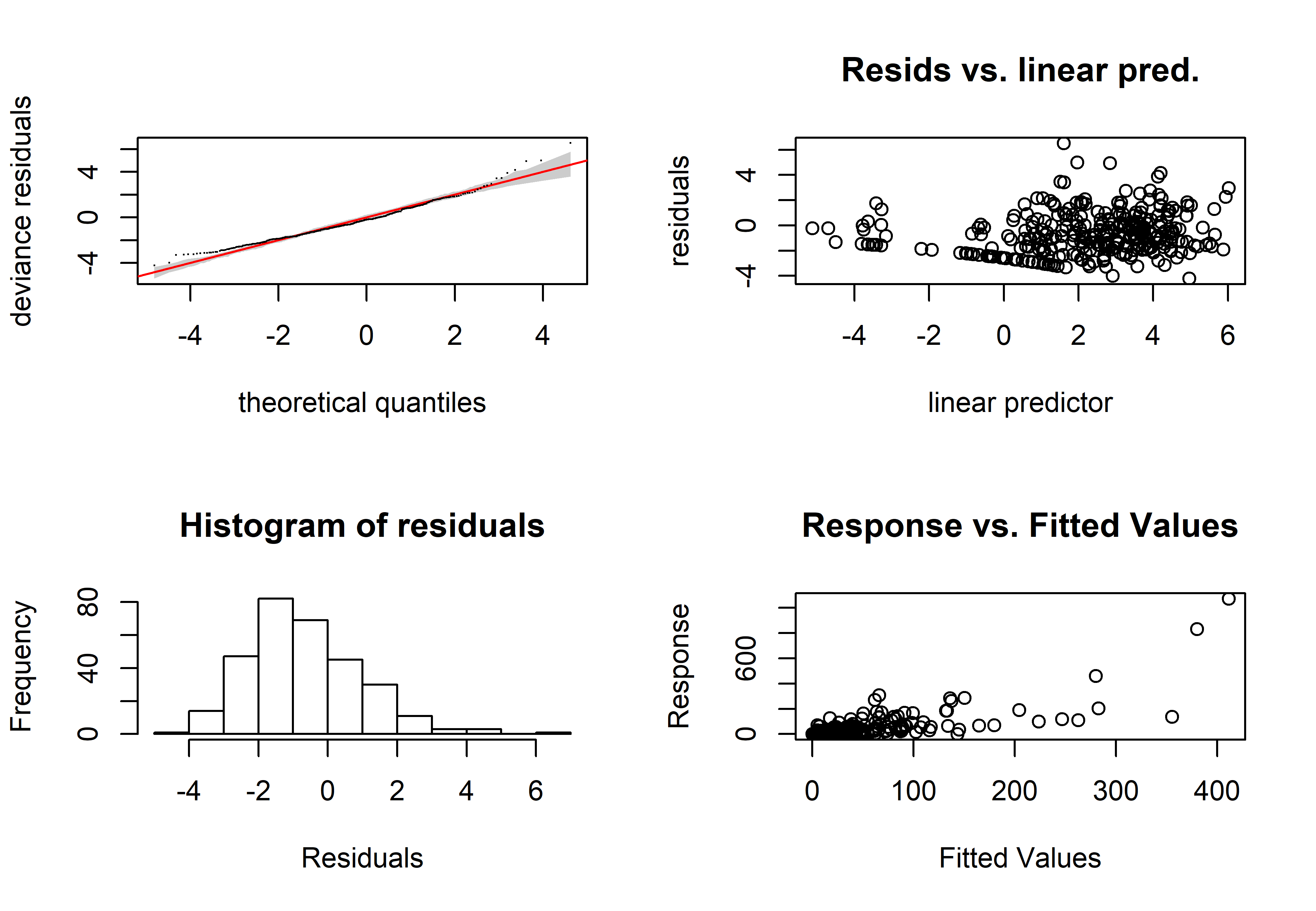

Supplement: Supplementary file 1 — Additional file 1. Species distribution methods and results to estimate receptivity + model diagnostics (all models). [file 12942_2020_241_MOESM1_ESM.zip › S10.png]

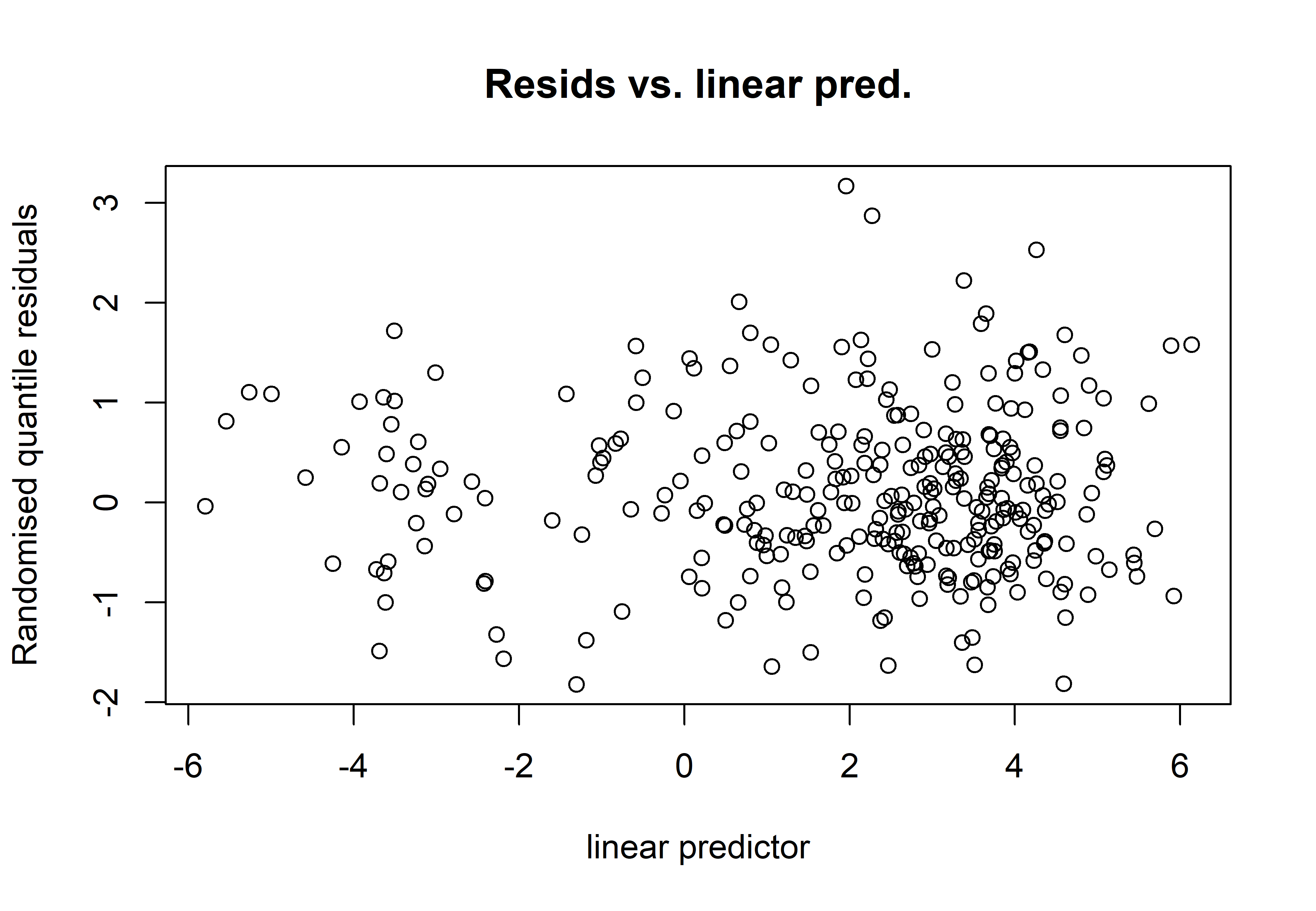

Supplement: Supplementary file 1 — Additional file 1. Species distribution methods and results to estimate receptivity + model diagnostics (all models). [file 12942_2020_241_MOESM1_ESM.zip › S11.png]

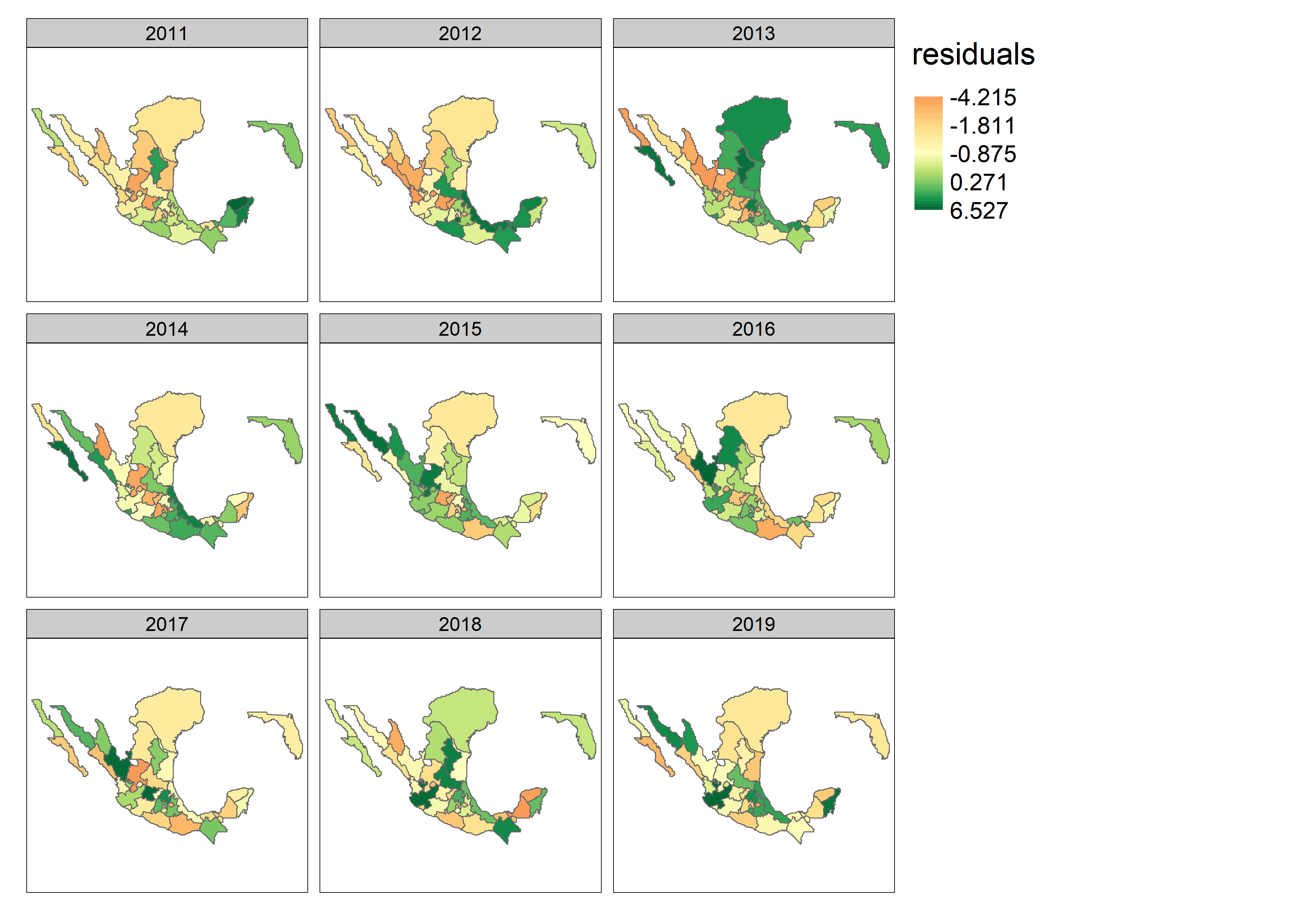

Supplement: Supplementary file 1 — Additional file 1. Species distribution methods and results to estimate receptivity + model diagnostics (all models). [file 12942_2020_241_MOESM1_ESM.zip › S12.png]

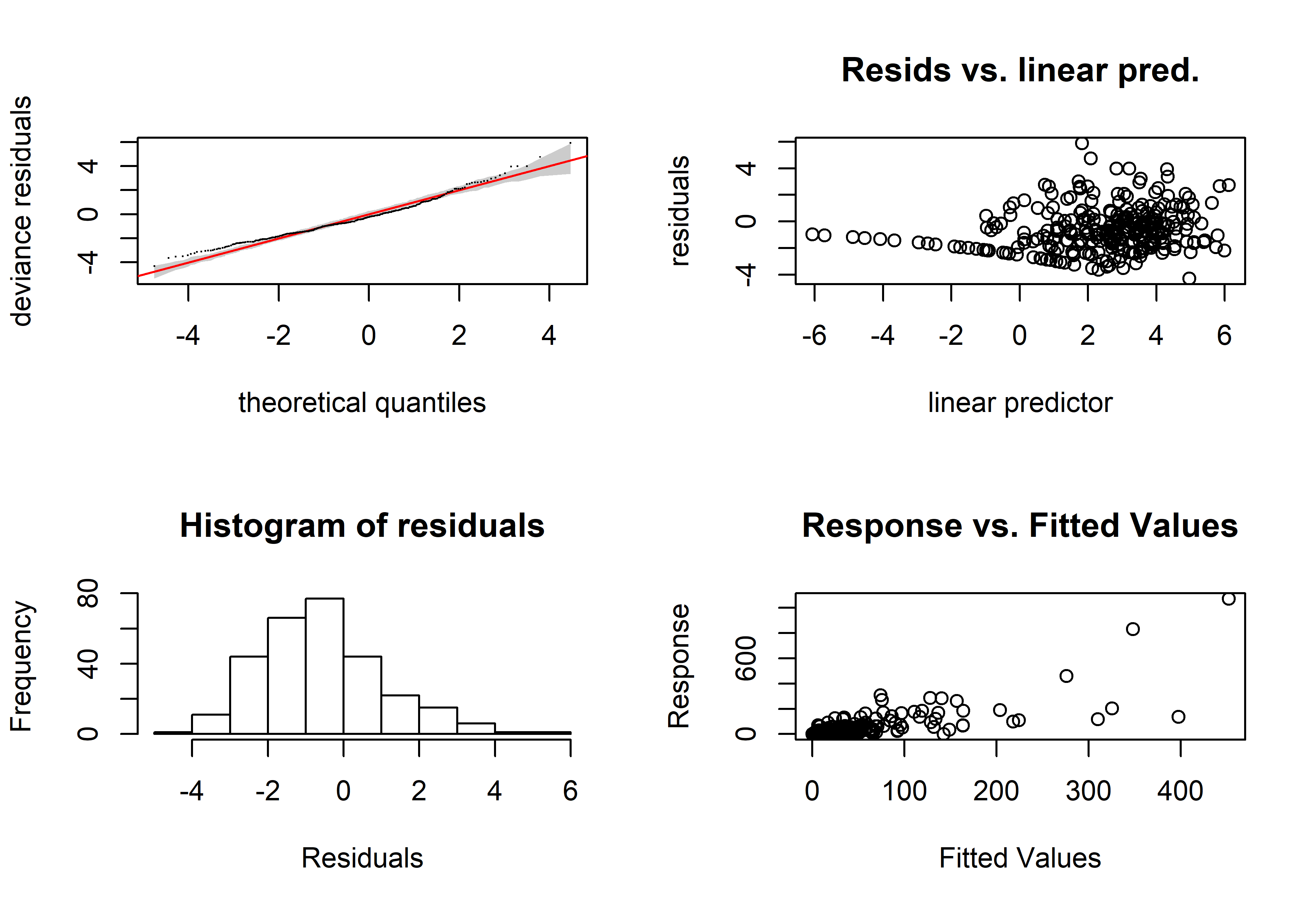

Supplement: Supplementary file 1 — Additional file 1. Species distribution methods and results to estimate receptivity + model diagnostics (all models). [file 12942_2020_241_MOESM1_ESM.zip › S13.png]

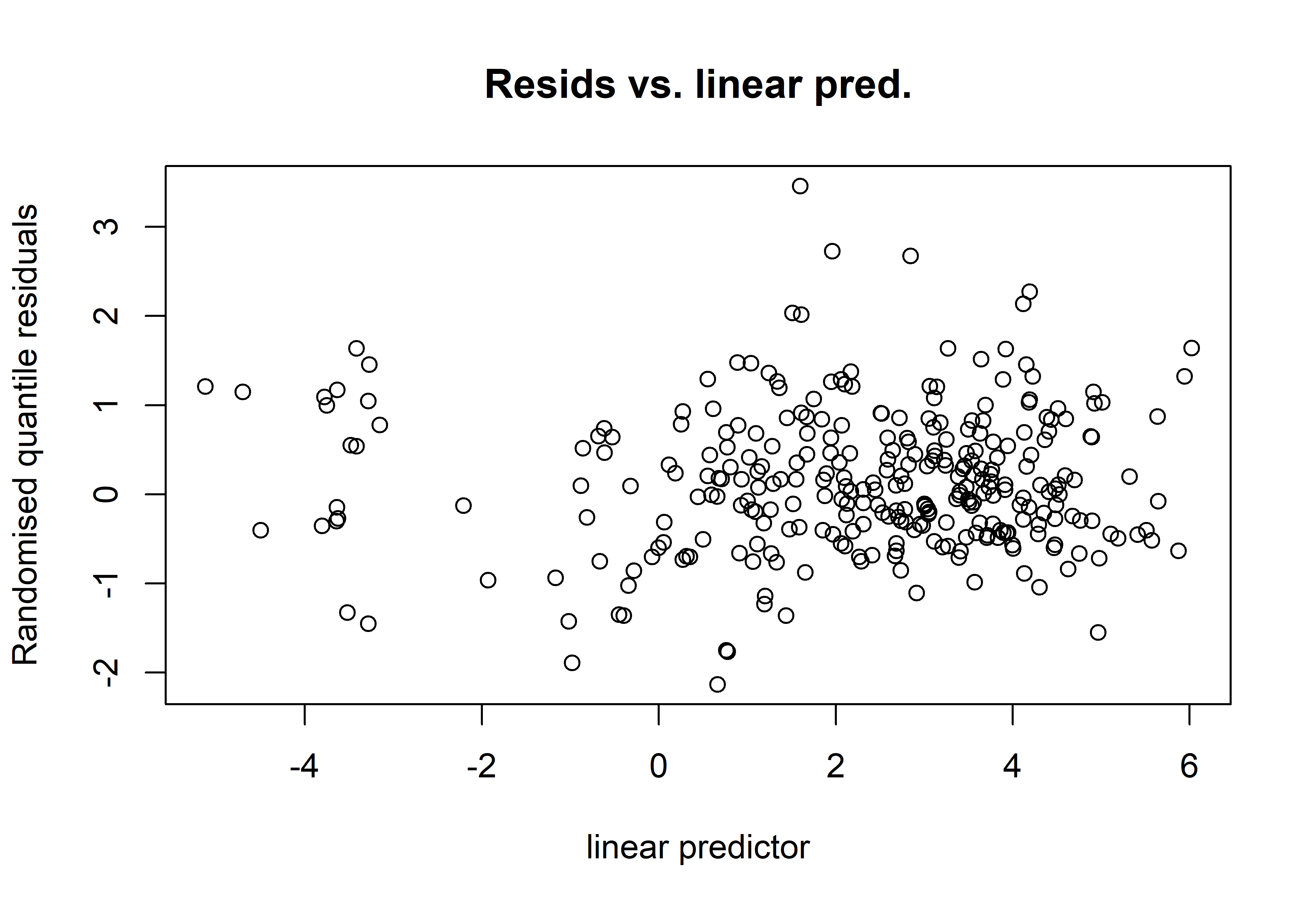

Supplement: Supplementary file 1 — Additional file 1. Species distribution methods and results to estimate receptivity + model diagnostics (all models). [file 12942_2020_241_MOESM1_ESM.zip › S14.png]

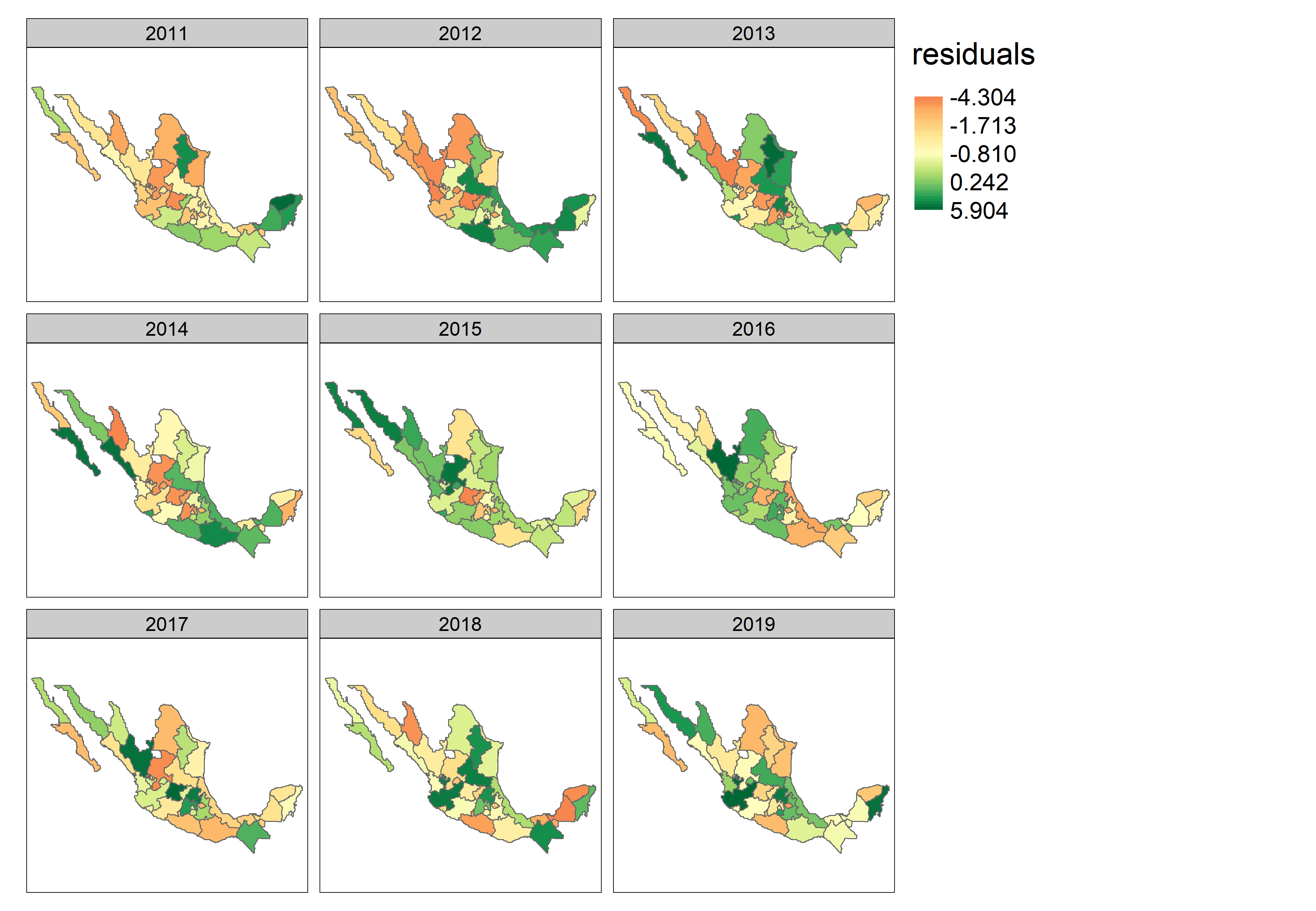

Supplement: Supplementary file 1 — Additional file 1. Species distribution methods and results to estimate receptivity + model diagnostics (all models). [file 12942_2020_241_MOESM1_ESM.zip › S15.png]

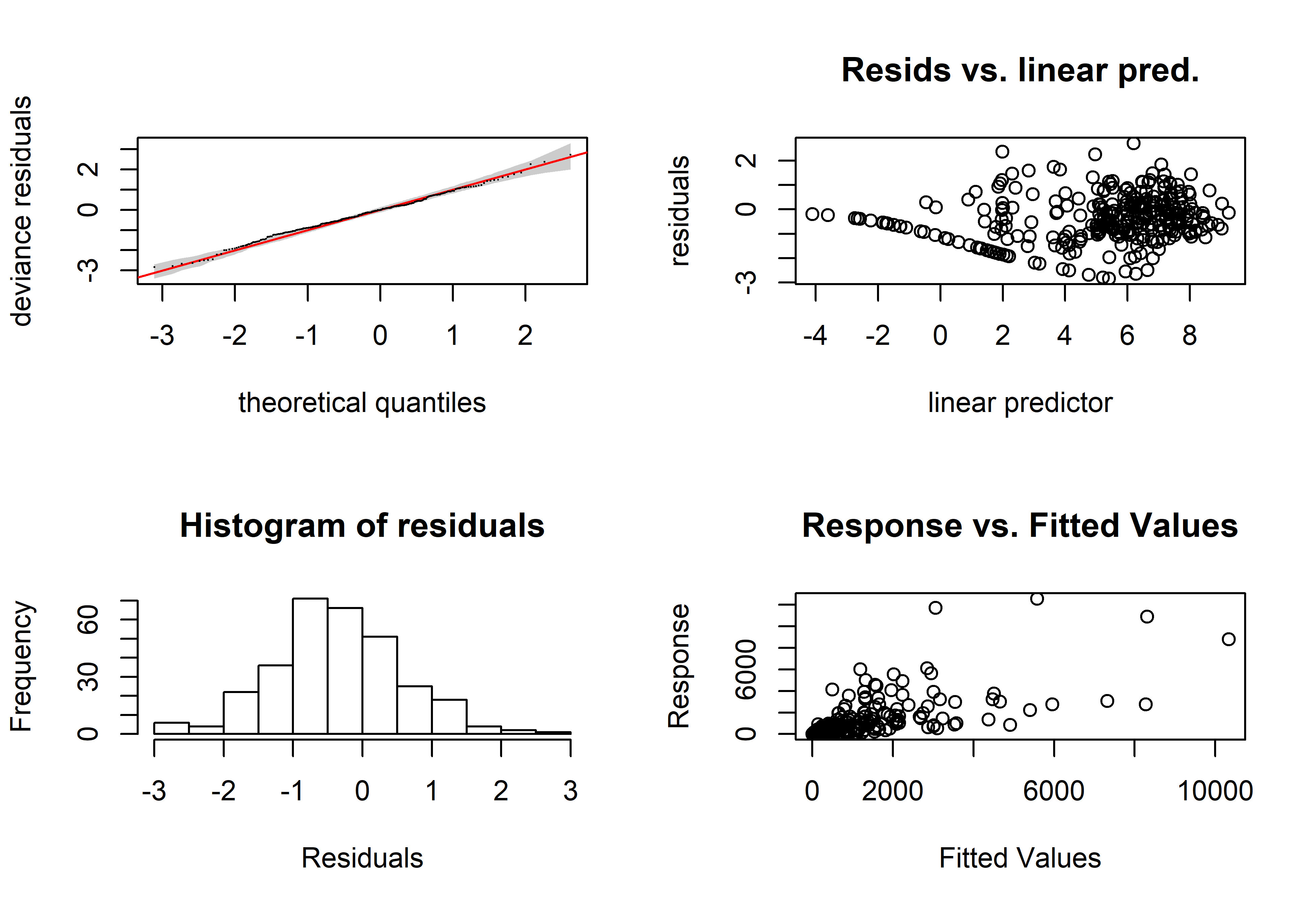

Supplement: Supplementary file 1 — Additional file 1. Species distribution methods and results to estimate receptivity + model diagnostics (all models). [file 12942_2020_241_MOESM1_ESM.zip › S16.png]

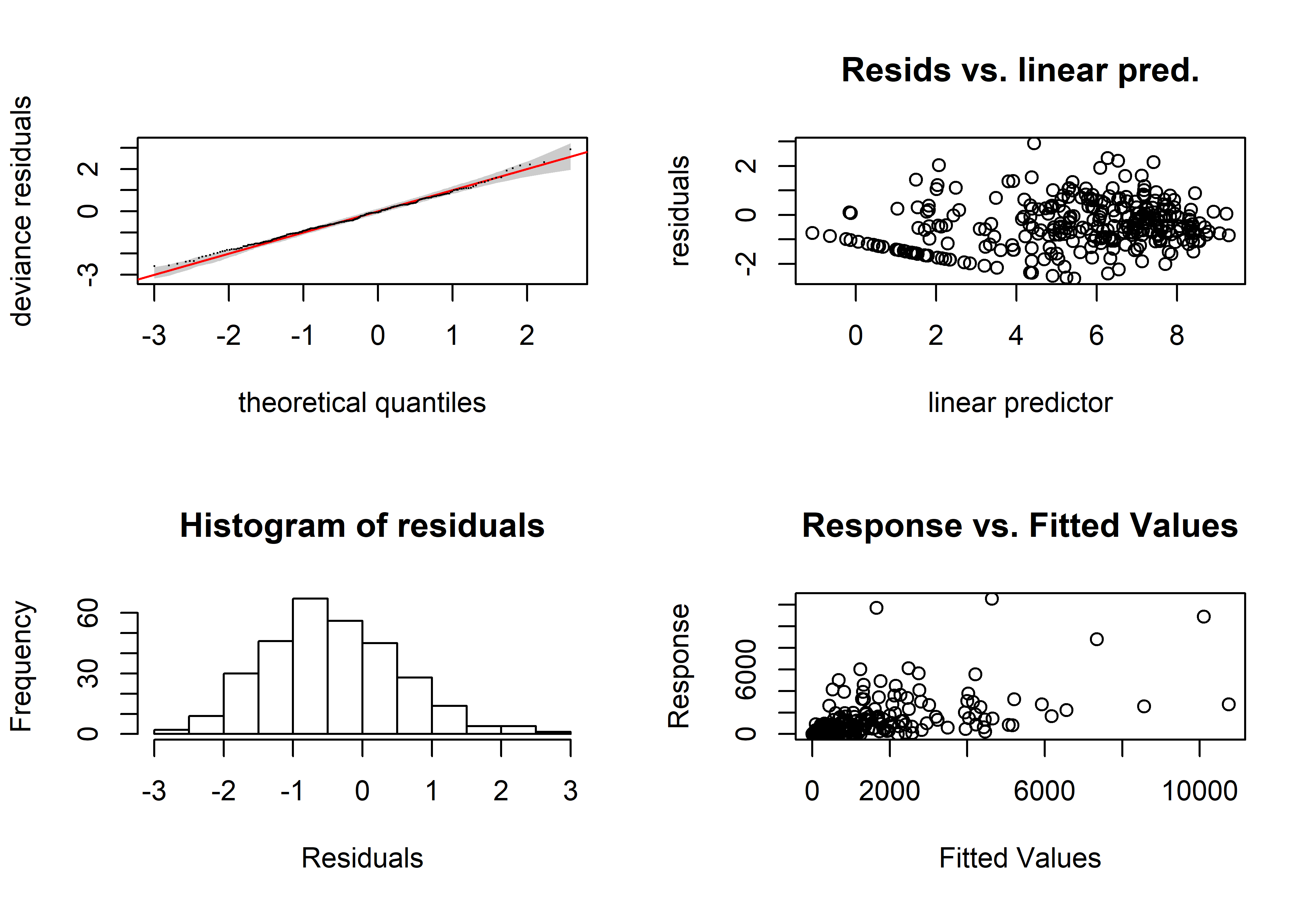

Supplement: Supplementary file 1 — Additional file 1. Species distribution methods and results to estimate receptivity + model diagnostics (all models). [file 12942_2020_241_MOESM1_ESM.zip › S17.png]

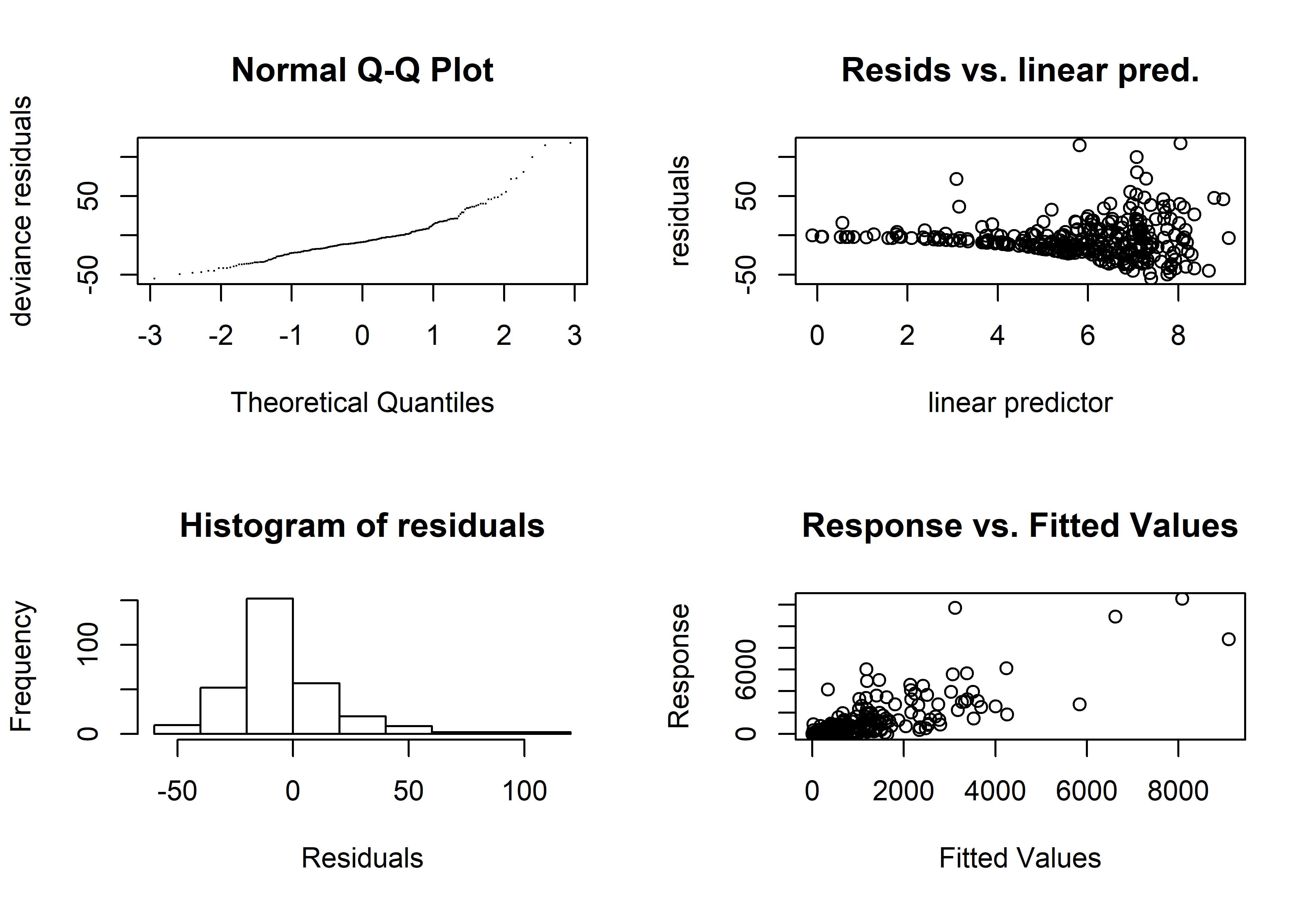

Supplement: Supplementary file 1 — Additional file 1. Species distribution methods and results to estimate receptivity + model diagnostics (all models). [file 12942_2020_241_MOESM1_ESM.zip › S18.png]

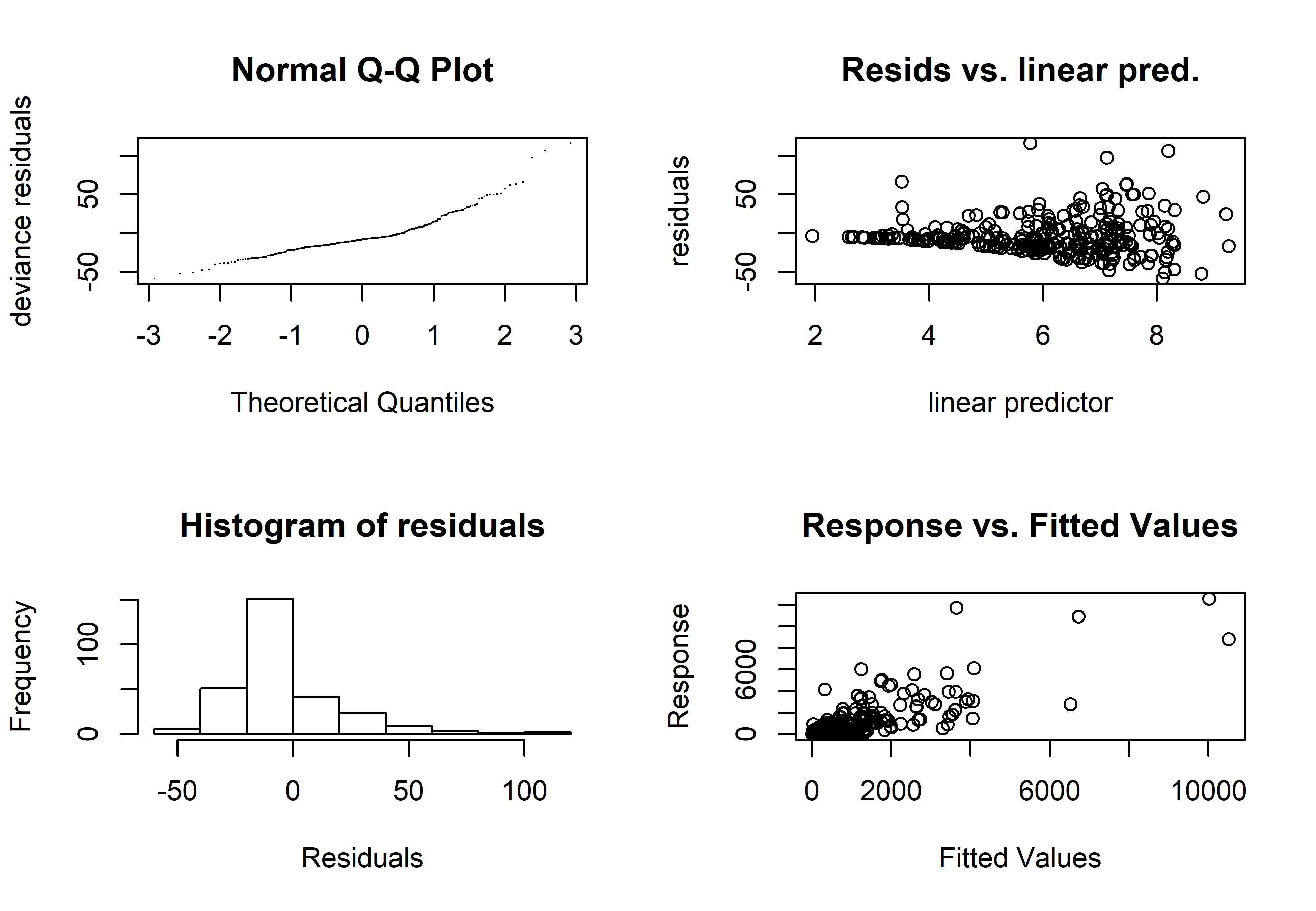

Supplement: Supplementary file 1 — Additional file 1. Species distribution methods and results to estimate receptivity + model diagnostics (all models). [file 12942_2020_241_MOESM1_ESM.zip › S19.png]

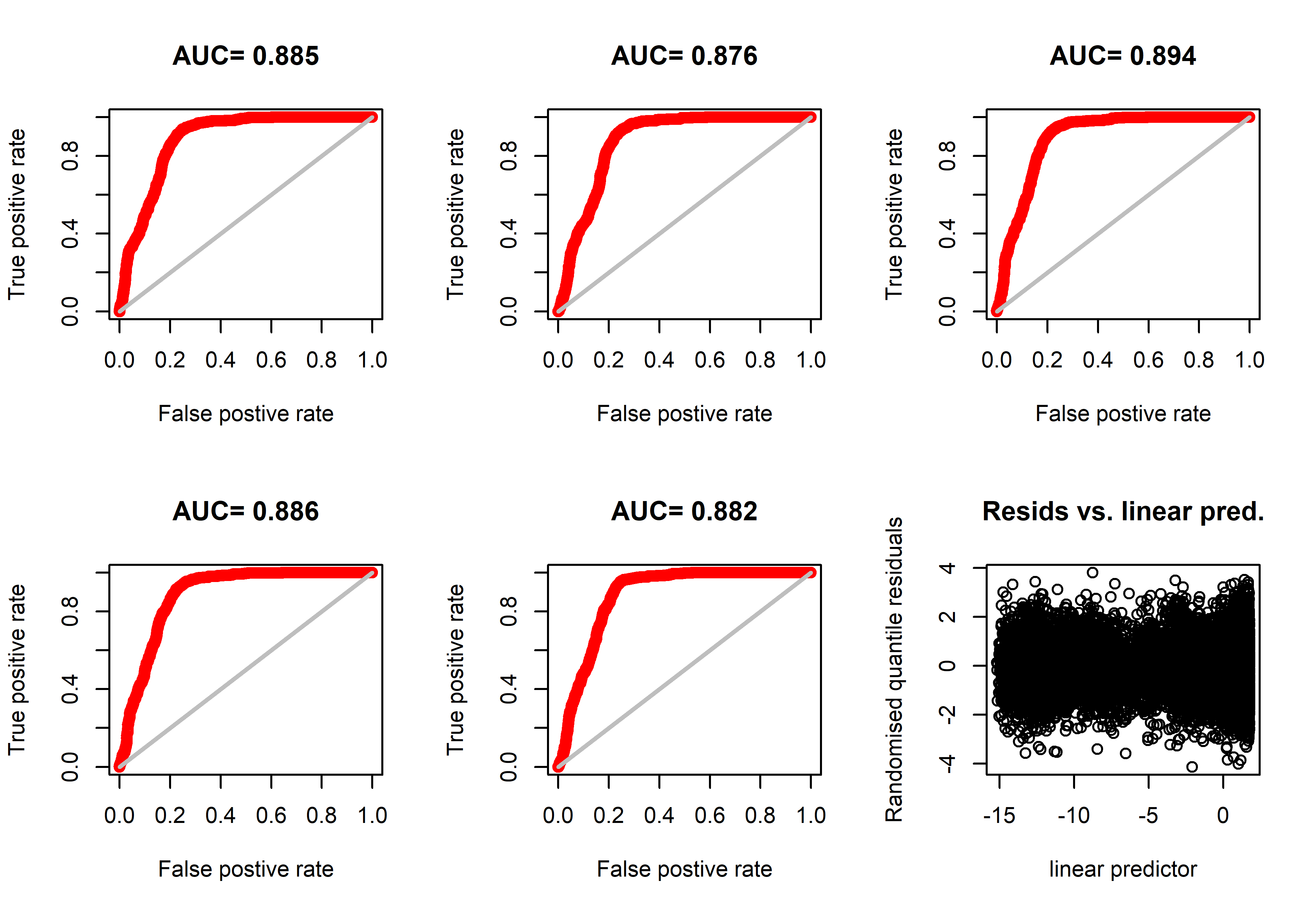

Supplement: Supplementary file 1 — Additional file 1. Species distribution methods and results to estimate receptivity + model diagnostics (all models). [file 12942_2020_241_MOESM1_ESM.zip › S6.png]

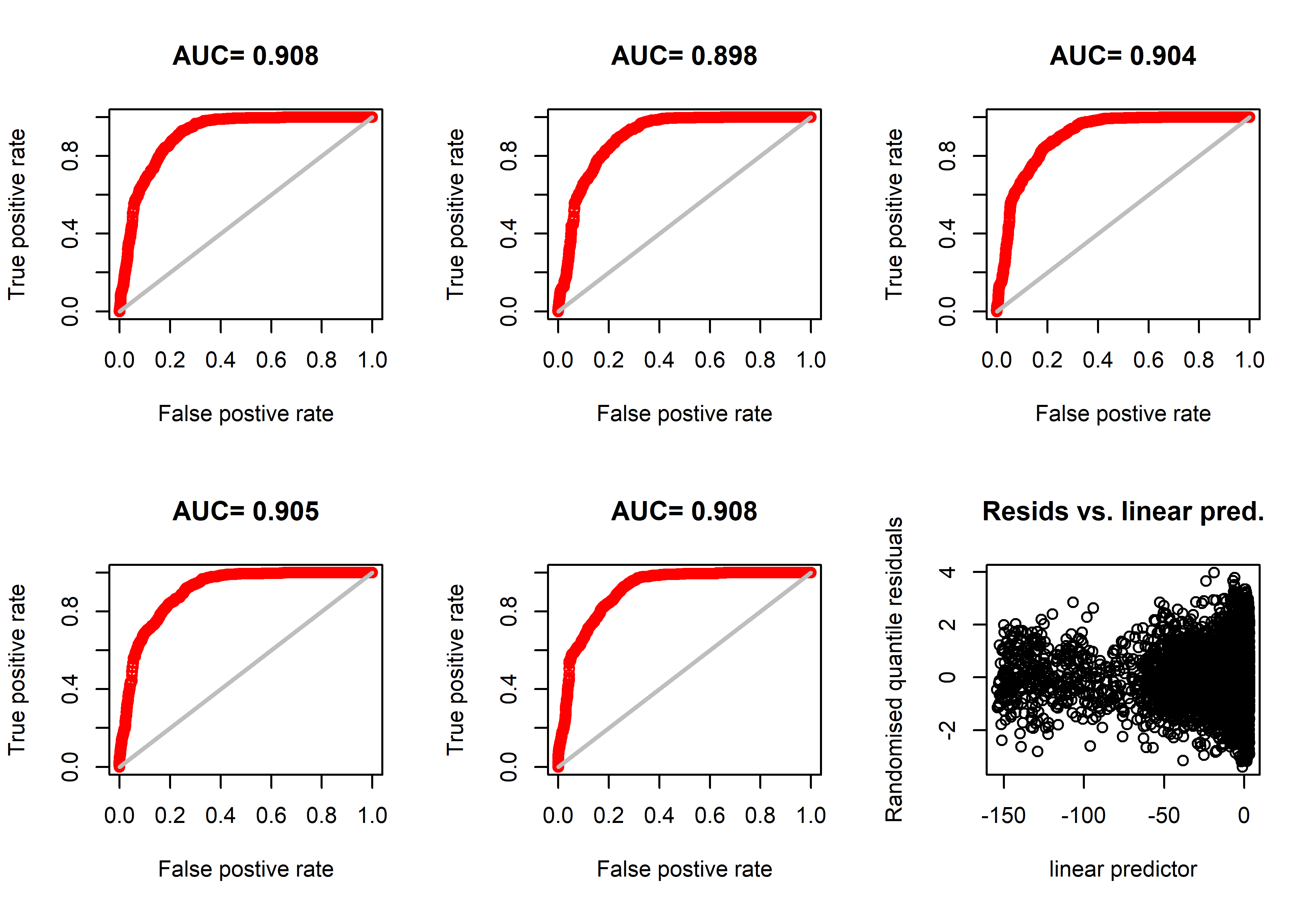

Supplement: Supplementary file 1 — Additional file 1. Species distribution methods and results to estimate receptivity + model diagnostics (all models). [file 12942_2020_241_MOESM1_ESM.zip › S7.png]

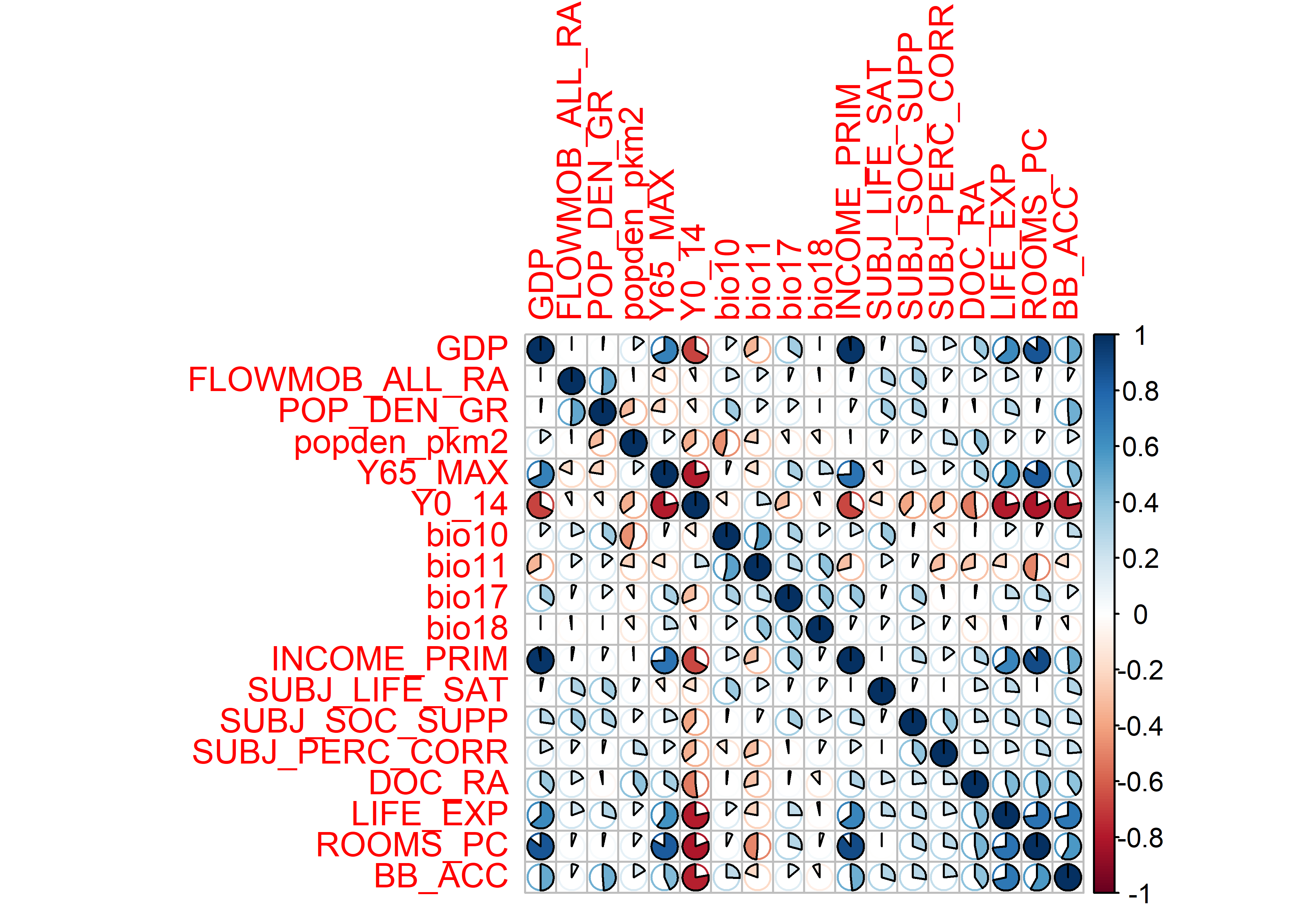

Supplement: Supplementary file 1 — Additional file 1. Species distribution methods and results to estimate receptivity + model diagnostics (all models). [file 12942_2020_241_MOESM1_ESM.zip › S8.png]

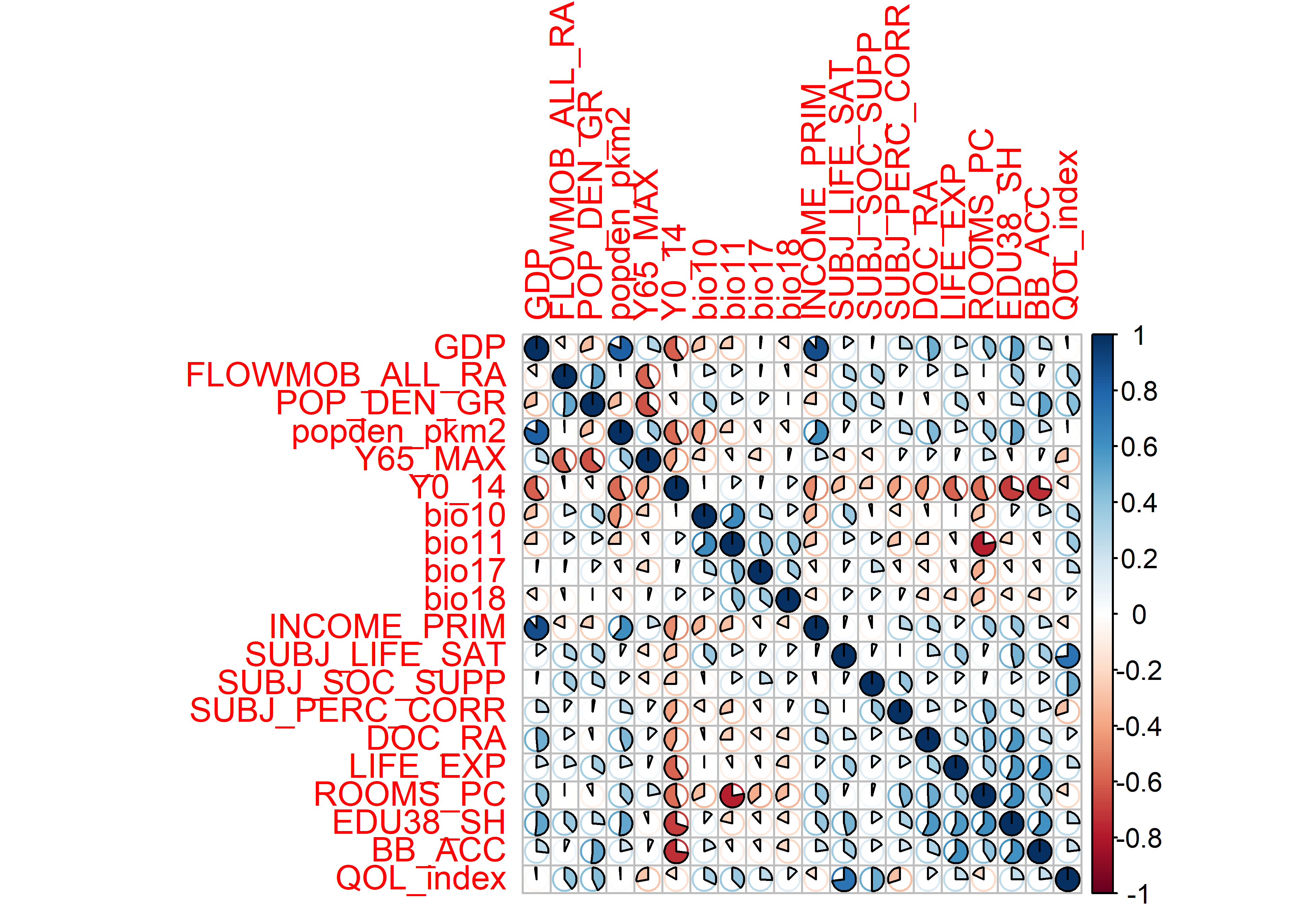

Supplement: Supplementary file 1 — Additional file 1. Species distribution methods and results to estimate receptivity + model diagnostics (all models). [file 12942_2020_241_MOESM1_ESM.zip › S9.png]
